# Antibody responses to SARS-CoV-2 vaccines in 45,965 adults from the general population of the United Kingdom

**DOI:** 10.1038/s41564-021-00947-3

**Published:** 2021-07-21

**Authors:** Jia Wei, Nicole Stoesser, Philippa C. Matthews, Daniel Ayoubkhani, Ruth Studley, Iain Bell, John I. Bell, John N. Newton, Jeremy Farrar, Ian Diamond, Emma Rourke, Alison Howarth, Brian D. Marsden, Sarah Hoosdally, E. Yvonne Jones, David I. Stuart, Derrick W. Crook, Tim E. A. Peto, Koen B. Pouwels, David W. Eyre, A. Sarah Walker, the COVID-19 Infection Survey team, Alex Lambert, Tina Thomas, Russell Black, Antonio Felton, Megan Crees, Joel Jones, Lina Lloyd, Esther Sutherland, Emma Pritchard, Karina-Doris Vihta, George Doherty, James Kavanagh, Kevin K. Chau, Stephanie B. Hatch, Daniel Ebner, Lucas Martins Ferreira, Thomas Christott, Wanwisa Dejnirattisai, Juthathip Mongkolsapaya, Sarah Cameron, Phoebe Tamblin-Hopper, Magda Wolna, Rachael Brown, Richard Cornall, Gavin Screaton, Katrina Lythgoe, David Bonsall, Tanya Golubchik, Helen Fryer, Stuart Cox, Kevin Paddon, Tim James, Thomas House, Julie Robotham, Paul Birrell, Helena Jordan, Tim Sheppard, Graham Athey, Dan Moody, Leigh Curry, Pamela Brereton, Ian Jarvis, Anna Godsmark, George Morris, Bobby Mallick, Phil Eeles, Jodie Hay, Harper VanSteenhouse, Jessica Lee

**Affiliations:** 1 Nuffield Department of Medicine, University of Oxford, Oxford, UK; 2 Big Data Institute, Nuffield Department of Population Health, University of Oxford, Oxford, UK; 3 The National Institute for Health Research Health Protection Research Unit in Healthcare Associated Infections and Antimicrobial Resistance at the University of Oxford, Oxford, UK; 4 The National Institute for Health Research Oxford Biomedical Research Centre, University of Oxford, Oxford, UK; 5 Department of Infectious Diseases and Microbiology, Oxford University Hospitals NHS Foundation Trust, John Radcliffe Hospital, Oxford, UK; 6 Office for National Statistics, Newport, UK; 7 Office of the Regius Professor of Medicine, University of Oxford, Oxford, UK; 8 Health Improvement Directorate, Public Health England, London, UK; 9 Wellcome Trust, London, UK; 10 Nuffield Department of Orthopaedics, Rheumatology and Musculoskeletal Sciences, University of Oxford, Oxford, UK; 11 Health Economics Research Centre, Nuffield Department of Population Health, University of Oxford, Oxford, UK; 12 MRC Clinical Trials Unit at UCL, UCL, London, UK; 13 Oxford University Hospitals NHS Foundation Trust, Oxford, UK; 14 University of Manchester, Manchester, UK; 15 IQVIA, London, UK; 16 National Biocentre, Milton Keynes, UK; 17 Glasgow Lighthouse Laboratory, London, UK; 18 Department of Health and Social Care, London, UK

**Keywords:** Diagnostic markers, Viral infection

## Abstract

We report that in a cohort of 45,965 adults, who were receiving either the ChAdOx1 or the BNT162b2 SARS-CoV-2 vaccines, in those who had no prior infection with SARS-CoV-2, seroconversion rates and quantitative antibody levels after a single dose were lower in older individuals, especially in those aged >60 years. Two vaccine doses achieved high responses across all ages. Antibody levels increased more slowly and to lower levels with a single dose of ChAdOx1 compared with a single dose of BNT162b2, but waned following a single dose of BNT162b2 in older individuals. In descriptive latent class models, we identified four responder subgroups, including a ‘low responder’ group that more commonly consisted of people aged >75 years, males and individuals with long-term health conditions. Given our findings, we propose that available vaccines should be prioritized for those not previously infected and that second doses should be prioritized for individuals aged >60 years. Further data are needed to better understand the extent to which quantitative antibody responses are associated with vaccine-mediated protection.

## Abstract (web summary)

Longitudinal tracing of antibody responses to the ChAdOx1 and the BNT162b2 COVID-19 vaccines in 45,965 adults from the United Kingdom give indications for vaccine prioritization.

## Main

Multiple vaccines have been developed that offer protection against COVID-19 by generating immune responses against the spike antigen of SARS-CoV-2. On 8 December 2020, the United Kingdom (UK) started its national vaccination programme with the Pfizer–BioNTech BNT162b2 vaccine^1^, followed by the approval of the Oxford–AstraZeneca ChAdOx1 nCoV-19 vaccine, first used outside a clinical trial on 4 January 2021 (ref. ^2^). Both vaccines have been widely used in the UK.

Vaccines were initially administered to priority groups, including care home residents, people >80 years old, healthcare workers and those clinically vulnerable (≥16 years), and then offered to the rest of the adult (≥18 years) population in decreasing age order^3^. To maximize initial coverage, in early January 2021, the dosing interval was extended to 12 weeks for all vaccines, regardless of the licensed dosing schedule. Up until 6 April 2021, 31.7 million people (60.2% of the population aged ≥18 years) have been given a first dose, and 5.7 million people (10.8%) have received two vaccine doses (https://coronavirus.data.gov.uk/details/vaccinations).

The efficacy of the ChAdOx1 and BNT162b2 vaccines against symptomatic laboratory-confirmed SARS-CoV-2 infection has been reported in large randomized controlled clinical trials as 52% (95% confidence interval (CI) = 30–86%) after the first dose and 95% (95% CI = 90–98%) after the second dose of BNT162b2 (ref. ^4^), and 70% (95% CI = 55–81%) after the second dose of ChAdOx1 (ref. ^5^). Several studies have examined the immunogenicity of vaccines in healthcare workers, who were typically the earliest groups to be vaccinated. A study of 3,610 healthcare workers found that 99.5% and 97.1% seroconverted after a single dose of BNT162b2 or ChAdOx1, respectively, and that higher quantitative immunoglobulin G (IgG) levels were achieved in previously infected individuals^6^. Other studies have also found that single-dose BNT162b2 elicited higher antibody levels in previously seropositive individuals, levels that were comparable to those after two doses of vaccines in seronegative individuals^7–9^. Outside trials, there are limited data on post-vaccine antibody responses in other groups, especially older adults who were underrepresented in the ChAdOx1 trial^5^. A study of 185 individuals aged >70 years showed high seropositivity after one or two BNT162b2 doses^10^. Another study, of 100 individuals aged 80–100 years, showed almost universal high antibody responses 3 weeks after a single dose of BNT162b2, with spike-specific cellular responses in 63% of participants^11^. However, the representativeness of these small cohorts is unclear.

Real-world data provide information on populations who may not participate in clinical trials and can be used to assess the efficacy of interventions as deployed. We used the UK’s national COVID-19 Infection Survey (ISRCTN21086382), which includes a representative sample of households and has longitudinal follow-up, to study population-wide anti-trimeric spike IgG antibody responses after SARS-CoV-2 vaccination by time since vaccination, considering the vaccine type (BNT162b2 or ChAdOx1), the number of doses received, the presence or absence of prior SARS-CoV-2 infection and demographic factors. Our results build on the REACT-2 study, a serial cross-sectional UK study of antibody responses using a binary point-of-care lateral flow assay^12^. Specifically, we investigate longitudinal data in the same individuals with a validated quantitative laboratory antibody assay, which has previously been shown to correlate with neutralizing activity (correlation coefficient of 0.76)^13^, allowing the assay to act as a potential correlate of protection based on the strong association between quantitative neutralizing activity and protection from infection^14^. Supporting this, quantitative readings from the assay are associated with protection from infection in those previously infected^15^.

## Results

**Fig. 1:**
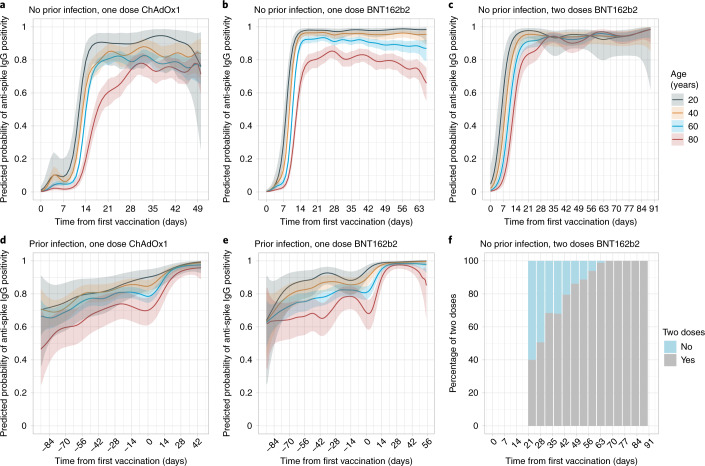
Predicted probability of anti-spike IgG positivity by time from first vaccination for comparisons of age by vaccine type and prior infection status. The data are from 40,131 participants without prior infection and 5,834 participants with prior infection. **a**, No prior infection and received one dose of ChAdOx1. **b**, No prior infection and received one dose of BNT162b2. **c**, No prior infection and received two doses of BNT162b2. **d**, With prior infection and received one dose of ChAdOx1. **e**, With prior infection and received one dose of BNT162b2. **f**, For those who received two doses of BNT162b2 without prior infection, the chart shows the percentage of participants having had two vaccine doses by each time point (grey, had two doses; blue, had only one dose). Different *x* axis scales reflect different durations of follow-up post-vaccination in the different cohorts. Line colour indicates antibody response predicted for ages 20, 40, 60 and 80 years (see Extended Data Fig. 2 for the full model across all ages and comparisons of vaccine type by age). The 95% CIs are calculated by prediction ± 1.96 × standard error of prediction. Source data

**Extended Data Fig. 1:**
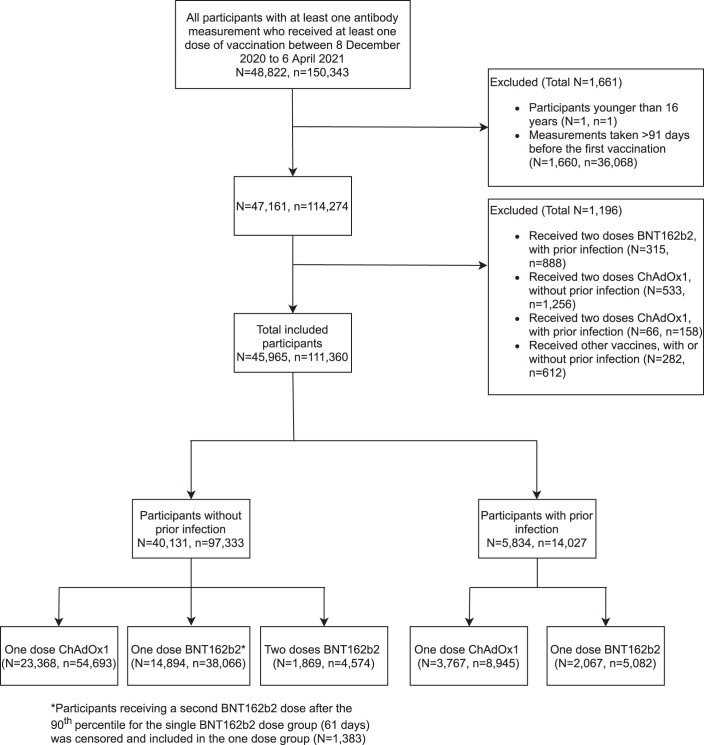
Flowchart of the study cohort. *N* represents the number of participants, and *n* represents the number of antibody measurements.

In all, 45,965 participants aged ≥16 years from the general population who were first vaccinated between 8 December 2020 and 6 April 2021 contributed a total of 111,360 SARS-CoV-2 anti-spike IgG measurements taken at any point between 91 days before the first vaccination date up until 6 April 2021 (Extended Data Fig. 1). The median (interquartile (IQR)) age was 64 (54–71) years, and 25,330 (55.1%) were female. A total of 2,745 (6.0%) were healthcare workers, and 15,334 (33.4%) had a long-term health condition (Supplementary Table 1). In all, 5,834 (12.7%) participants with a SARS-CoV-2 PCR-positive study nose/throat swab or anti-spike IgG-positive study antibody result at any time before vaccination were considered to have been previously infected with SARS-CoV-2, irrespective of whether they had reported previous symptoms or not. Using this definition, 3,767 (8.2%) and 2,067 (4.5%) previously infected participants then received one dose of ChAdOx1 or BNT162b2, respectively. A total of 23,368 (50.8%), 14,894 (32.4%) and 1,869 (4.1%) participants without evidence of prior infection received one dose of ChAdOx1, one dose of BNT162b2 or two doses of BNT162b2, respectively. Among 1,869 (4.1%) participants without evidence of prior infection who received two doses of BNT162b2, the median (IQR) duration between two doses was 31 (21–47) days, with 1,020 (54.6%) ≤31 days (Fig. 1f and Supplementary Table 1). Participant characteristics varied across the different vaccination groups, which generally reflected vaccine prioritization, with proportionately more healthcare workers and the oldest individuals having received two doses of BNT162b2.

### Antibody positivity after vaccination

**Extended Data Fig. 2:**
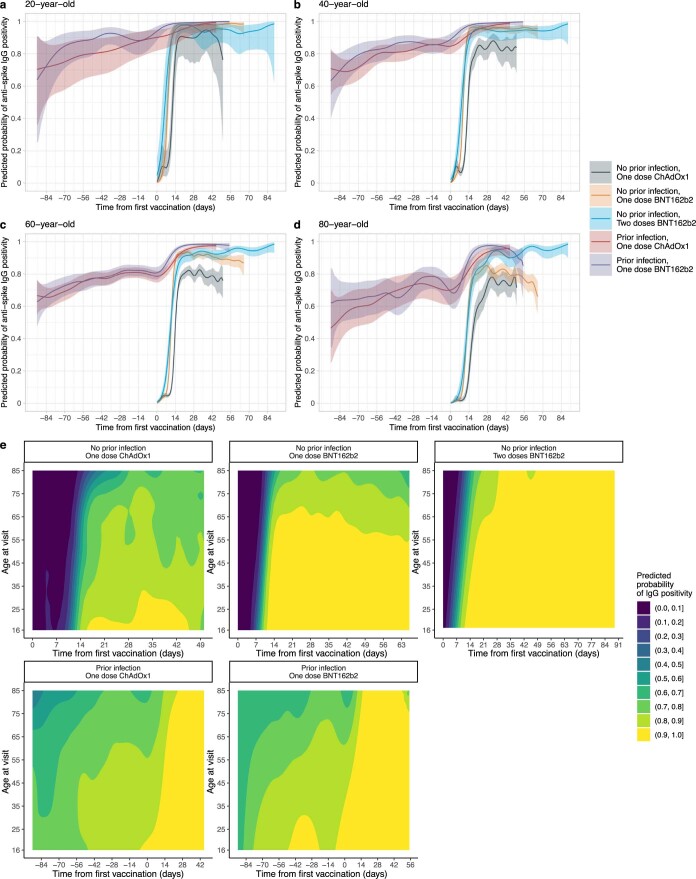
Predicted probability of anti-spike IgG positivity by time from first vaccination based on data from 40,131 participants without prior infection and 5,834 participants with prior infection. **a**, 20-year-old. **b**, 40-year-old. **c**, 60-year-old. **d**, 80-year-old. Line colour indicates different vaccine type and prior infection status. The 95% confidence intervals are calculated by prediction ± 1.96*standard error of the prediction. Data identical to Fig. 1, but Fig. 1 panels represent age rather than vaccine type as here. **e**, Predicted probability of anti-spike IgG positivity by time from first vaccination and age, according to vaccine type and prior infection status (full model). Predictions shown for specific ages in Fig. 1. Observed data in Extended Data Fig. 3–7. Source data

**Extended Data Fig. 3:**
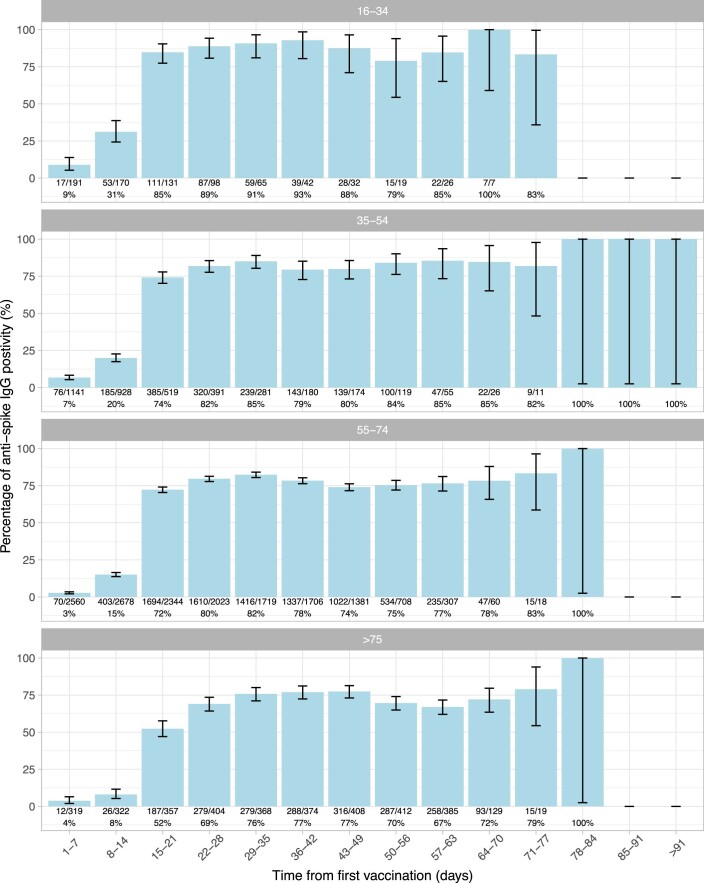
Percentage (95% CI) anti-spike IgG positive by days after first vaccination in 23,368 participants receiving a single dose of ChAdOx1 vaccine and without evidence of prior infection. Results were divided into four age groups: 16–34, 35–54, 55–74 and >75 years. Exact binomial test was performed to obtain the 95% confidence intervals (shown as ‘error bars’). Numbers above the x-axis represent positive number/total number, and the percentage of positivity (%). Source data

**Extended Data Fig. 4:**
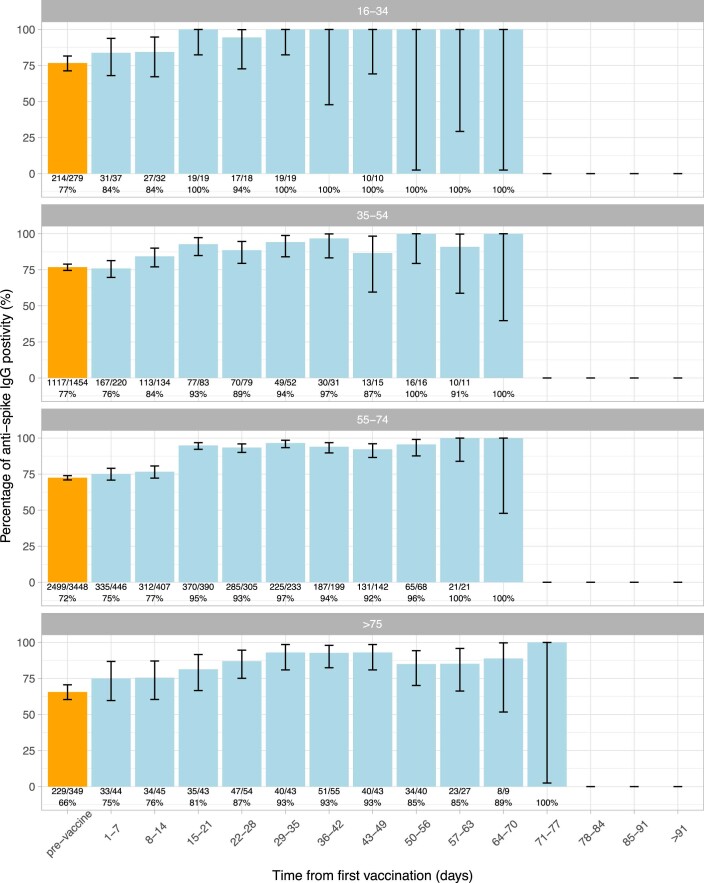
Percentage (95% CI) anti-spike IgG positives by days after first vaccination in 3,767 participants receiving a single dose of ChAdOx1 vaccine and with evidence of prior infection. Results were divided into four age groups: 16–34, 35–54, 55–74 and >75 years. Exact binomial test was performed to obtain the 95% confidence intervals (shown as ‘error bars’). Numbers above the *x*-axis represent positive number/total number, and the percentage of positivity (%). Source data

**Extended Data Fig. 5:**
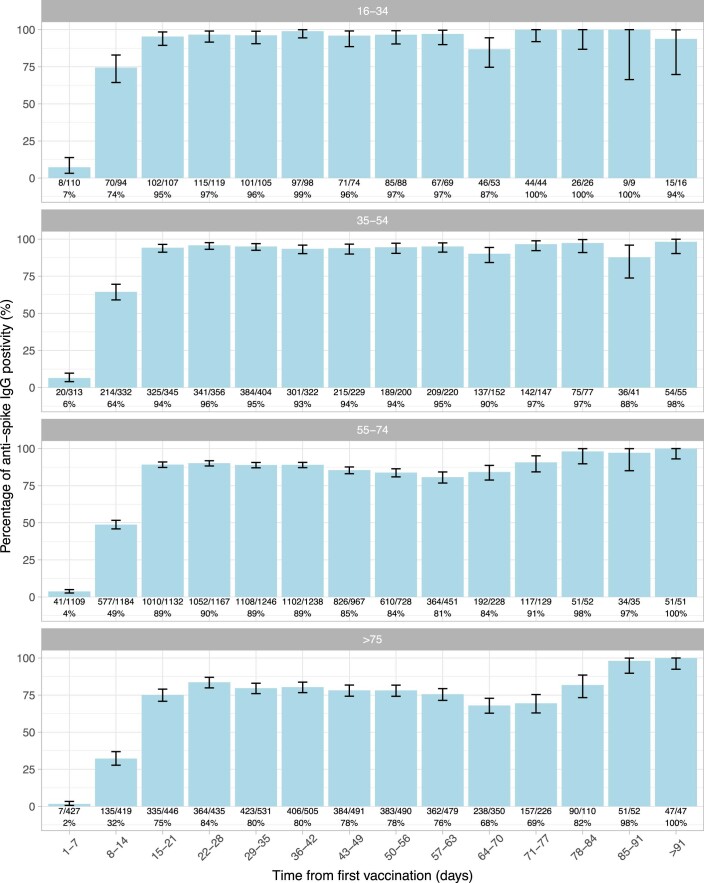
Percentage (95% CI) anti-spike IgG positives by days after first vaccination in 14,894 participants receiving a single dose of BNT162b2 vaccine and without evidence of prior infection. Results were divided into four age groups: 16–34, 35–54, 55–74 and >75 years. Exact binomial test was performed to obtain the 95% confidence intervals (shown as ‘error bars’). Numbers above the x-axis represent positive number/total number, and the percentage of positivity (%). Source data

**Extended Data Fig. 6:**
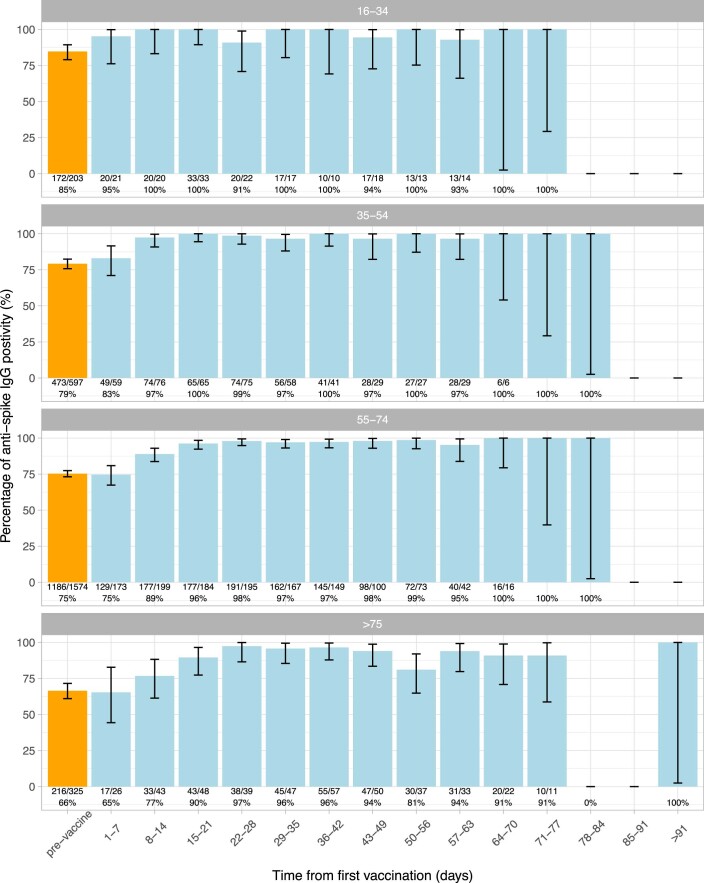
Percentage (95% CI) anti-spike IgG positives by days after first vaccination in 2,067 participants receiving a single dose of BNT162b2 vaccine and with evidence of prior infection. Results were divided into four age groups: 16–34, 35–54, 55–74 and >75 years. Exact binomial test was performed to obtain the 95% confidence intervals (shown as ‘error bars’). Numbers above the x-axis represent positive number/total number, and the percentage of positivity (%). Source data

**Extended Data Fig. 7:**
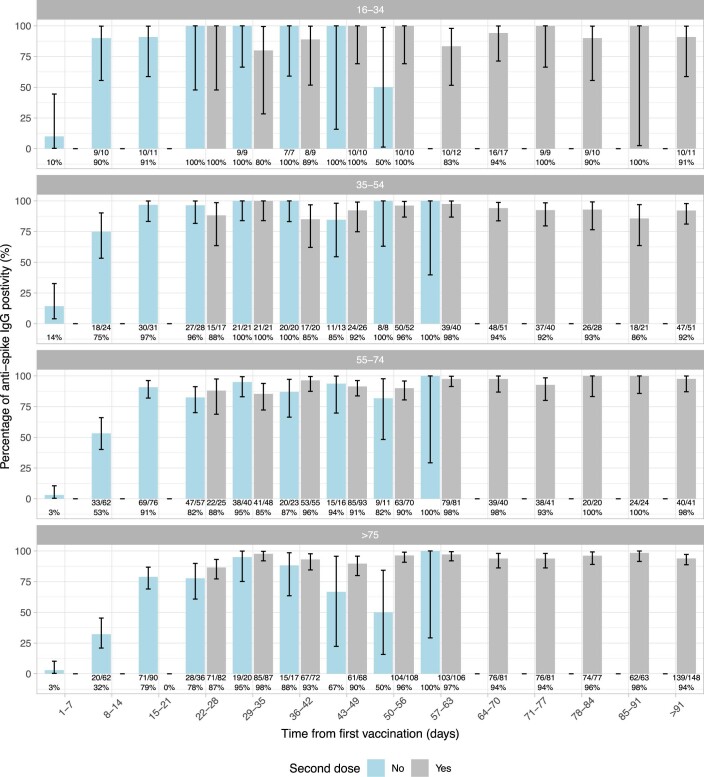
Percentage (95% CI) anti-spike IgG positives by days after first vaccination in 1,869 participants receiving two doses of BNT162b2 vaccine and without evidence of prior infection. Results were divided into four age groups: 16–34, 35–54, 55–74 and >75 years. Exact binomial test was performed to obtain the 95% confidence intervals (shown as ‘error bars’). Numbers above the x-axis represent positive number/total number, and the percentage of positivity (%). Source data

In participants without evidence of prior infection, models of binary (positive versus negative) post-vaccine antibody responses showed that positive anti-spike IgG results increased over the 2–4 weeks after the first vaccination and varied significantly by age (Fig. 1 and Extended Data Fig. 2, with observed numbers/percentages in Extended Data Figs. 3–7). Fewer older participants were seropositive after receiving a single dose of ChAdOx1 or BNT162b2. For example, the estimated percentage of seropositive 80 year olds was 74% (95% CI = 66–80%) and 85% (95% CI = 80–89%) 28 days after the first vaccination with ChAdOx1 or BNT162b2, respectively, compared with 79% (95% CI = 75–83%) and 91% (95% CI = 89–93%), respectively, for 60 year olds and 84% (95% CI = 76–89%) and 95% (95% CI = 92–97%), respectively, for 40 year olds (Supplementary Table 2). In contrast, two doses of BNT162b2 achieved >90% seropositivity 28–72 days after the first vaccination regardless of age, although there was some evidence of waning in those only receiving a first BNT162b2 dose at older ages. There was no evidence of differences in seropositivity rates 14–42 days after the first vaccine in those of younger ages (for example, 20 and 40 years) receiving one dose or two doses of BNT162b2, but greater rates of seroconversion were seen in older individuals (for example, 80 years) receiving two doses (Extended Data Fig. 2). There was no evidence of seropositivity declines following the first vaccine dose in older individuals receiving a single dose of ChAdOx1.

In participants with prior evidence of infection, before vaccination, younger participants were more likely to be seropositive. For example, on the day of vaccination 90% (95% CI = 82–95%) for 20 year olds, 85% (95% CI = 80–88%) for 40 year olds, 78% (95% CI = 75–82%) for 60 year olds and 70% (95% CI = 61–78%) for 80 year olds receiving ChAdOx1 were seropositive (Supplementary Table 2; the same trend occurred for BNT162b2). A high percentage of participants achieved positive antibody responses 28 days after vaccination (≥94%) regardless of age and the vaccine given, and the rate was similar to the positivity rate in participants without prior infection who received two doses of BNT162b2 (Extended Data Fig. 2).

### Associations with initial antibody response in those without evidence of prior infection

**Fig. 2:**
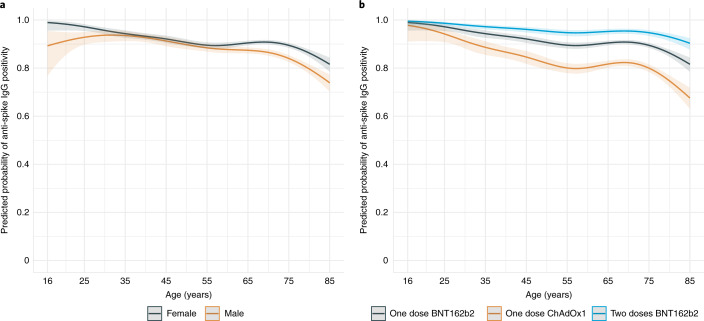
Predicted probability of anti-spike IgG positivity after first vaccination in participants without evidence of prior infection from a multivariable logistic regression model. Predicted probability with 95% CIs of anti-spike IgG positivity 14–60 days after first vaccination in 24,977 participants without evidence of prior infection. **a**, Predicted probability of anti-spike IgG positivity by age and sex. **b**, Predicted probability of anti-spike IgG positivity by age and vaccine type. Age was fitted using natural cubic spline with four internal knots placed at the 20th, 40th, 60th and 80th percentile (30, 44, 57 and 71 years) and two boundary knots at the 5th and 95th percentile (19 and 82 years). The 95% CIs were calculated by prediction ± 1.96 × standard error of the prediction and are shown as the shaded area. Testing for an interaction between sex and age was performed using a likelihood ratio test (*P* = 0.02). Values are plotted at the reference category for other variables (BNT162b2 one dose (**a**)/female (**b**), white ethnicity, index of multiple deprivation = 55, household size = 1, did not work in patient-facing healthcare or social care, did not work in a care home, no long-term health condition). Source data

A total of 28,144 participants had an anti-spike IgG measurement 14–60 days after their first ChAdOx1 or BNT162b2 vaccination, of whom 24,977 (88.7%) had no evidence of prior infection and were included in a separate logistic regression analysis to investigate associations with antibody positivity. In all, 20,505 (82.1%) had a positive post-vaccine anti-spike IgG result. Age, sex, vaccine type, ethnicity, social deprivation, healthcare roles and long-term health conditions were associated with seropositivity after vaccination (Table 1). Consistent with the data presented in Fig. 1, anti-spike IgG positivity decreased with older age, and the association was nonlinear, with seropositivity dropping faster for those aged >75 years (Fig. 2a,b). There was evidence of effect modification between age and sex, whereby at younger ages (30–55 years), similar rates of seroconversion were seen in males and females (for example, in 40 year olds, adjusted odds ratio (aOR) = 0.91 [95% CI = 0.68–1.22]), but at older ages (>60 years) males were less likely to seroconvert (for example, aOR = 0.65 [0.59–0.72] for 70 year olds) (Fig. 2a; interaction *P* = 0.02). Seroconversion by 60 days was less common following ChAdOx1 than after BNT162b2 vaccination (aOR = 0.47 [95% CI = 0.44–0.51]), while receiving two doses of BNT162b2 increased seroconversion compared with one BNT162b2 dose (aOR = 2.11 [1.69–2.66]). Patient-facing healthcare workers were more likely to be anti-spike IgG positive by 60 days post-vaccination (aOR = 1.63 [1.29–2.08]), and participants who had long-term health conditions were less likely (aOR = 0.64 [0.60–0.69]). There was evidence of greater seropositivity post-vaccination in participants from non-white ethnic groups (aOR = 1.54 [1.27–1.90]). A 10-unit increase in deprivation percentile (that is, decrease in deprivation) resulted in higher seropositivity post-vaccination (aOR = 1.28 [1.13–1.46]). There was no evidence of independent associations between antibody positivity and household size or working in social care or long-term care facilities.

**Table 1:** Predictors of antibody positivity 14–60 days post first vaccination in participants without evidence of prior infection from univariable and multivariable logistic regression models

	14–60 days post vaccination			Univariable			Multivariable		
	Negative (*N* = 4,472)	Positive (*N* = 20,505)	*P* value	OR	95% CI	*P* value	OR	95% CI	*P* value
Age^a^			<0.001			<0.001			
Median	69	67							
IQR	62, 74	58, 73							
Sex^a^			<0.001						
Female	2,085 (46.6%)	11,726 (57.2%)		1 (ref)					
Male	2,387 (53.4%)	8,779 (42.8%)		0.65	0.61–0.70	<0.001			
Vaccine type			<0.001						
One dose BNT162b2	1,323 (29.6%)	9,141 (44.6%)		1 (ref)			1 (ref)		
One dose ChAdOx1	3,058 (68.4%)	10,231 (49.9%)		0.48	0.45–0.52	<0.001	0.47	0.44–0.51	<0.001
Two doses BNT162b2	91 (2.0%)	1,133 (5.5%)		1.80	1.45–2.26	<0.001	2.11	1.69–2.66	<0.001
Ethnicity			<0.001						
White	4,356 (97.4%)	19,529 (95.2%)		1 (ref)			1 (ref)		
Non-white	116 (2.6%)	976 (4.8%)		1.88	1.55–2.29	<0.001	1.54	1.27–1.90	<0.001
Household size			<0.001						
1	1,160 (25.9%)	4,704 (22.9%)		1 (ref)			1 (ref)		
2	2,621 (58.6%)	11,900 (58.0%)		1.12	1.04–1.21	0.004	1.10	1.02–1.19	0.02
3	405 (9.1%)	2,140 (10.4%)		1.30	1.15–1.48	<0.001	1.03	0.90–1.17	0.7
4	202 (4.5%)	1,217 (5.9%)		1.49	1.27–1.75	<0.001	0.96	0.80–1.14	0.6
5+	84 (1.9%)	544 (2.7%)		1.60	1.27–2.04	<0.001	1.01	0.79–1.31	0.9
Deprivation percentile			0.001						
Median	63	64		(10-unit increase)			(10-unit increase)		
IQR	37, 82	40, 83		1.22	1.08–1.38	0.001	1.28	1.13–1.46	<0.001
Report working in patient-facing healthcare			<0.001						
No	4,386 (98.1%)	19,249 (93.9%)		1 (ref)			1 (ref)		
Yes	86 (1.9%)	1,256 (6.1%)		3.33	2.69–4.18	<0.001	1.63	1.29–2.08	<0.001
Report working in person-facing social care			<0.001						
No	4,427 (99.0%)	20,154 (98.3%)		1 (ref)			1 (ref)		
Yes	45 (1.0%)	351 (1.7%)		1.71	1.27–2.37	<0.001	1.00	0.72–1.40	1
Report working in care home (any role)			<0.001						
No	4,445 (99.4%)	20,177 (98.4%)		1 (ref)			1 (ref)		
Yes	27 (0.6%)	328 (1.6%)		2.68	1.84–4.06	<0.001	1.24	0.82–1.93	0.3
Report having long-term health condition			<0.001						
No	2,404 (53.8%)	13,427 (65.5%)		1 (ref)			1 (ref)		
Yes	2,068 (46.2%)	7,078 (34.5%)		0.61	0.57–0.65	<0.001	0.64	0.60–0.69	<0.001

^a^The combined effects of age and sex for the multivariable model are shown in Fig. 2. The 95% CIs are calculated by prediction ± 1.96 × standard error of the prediction; Wald *P* values are shown. Ref, reference category.

### Quantitative antibody response after vaccination

**Fig. 3:**
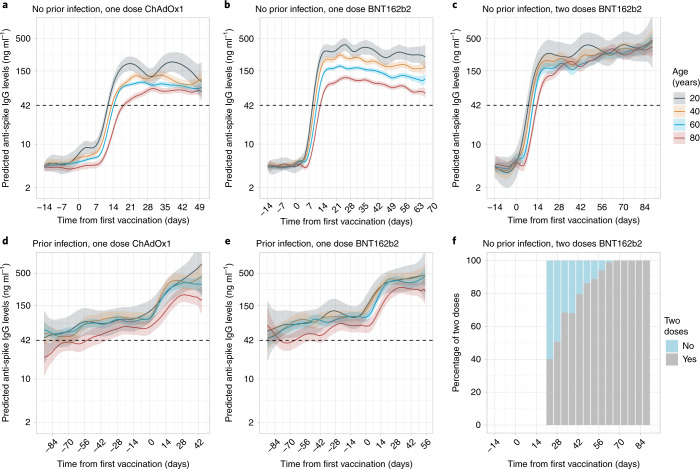
Predicted anti-spike IgG levels by time from first vaccination for comparisons of age by vaccine type and prior infection status. Predicted anti-spike IgG levels (mAb45 ng ml^–1^ equivalent units) by time from first vaccination based on data from 40,131 participants without prior infection and 5,834 participants with prior infection. **a**, No prior infection and received one dose of ChAdOx1. **b**, No prior infection and received one dose of BNT162b2. **c**, No prior infection and received two doses of BNT162b2. **d**, With prior infection and received one dose of ChAdOx1. **e**, With prior infection and received one dose of BNT162b2. **f**, For those who received two doses of BNT162b2 without prior infection, the chart shows the percentage of participants having had two vaccine doses by each time point (grey, had two doses; blue, had only one dose). Different *x* axis scales reflect different durations of follow-up post-vaccination in the different cohorts. Predicted levels are plotted on a log scale. Black dotted line indicates the threshold of IgG positivity (42 ng ml^–1^). Line colour indicates response predicted for ages 20, 40, 60 and 80 years (see Extended Data Fig. 8 for all ages and Fig. 4 for comparisons of vaccine type by age). The 95% CIs are calculated by prediction ± 1.96 × standard error of the prediction. Source data

**Fig. 4:**
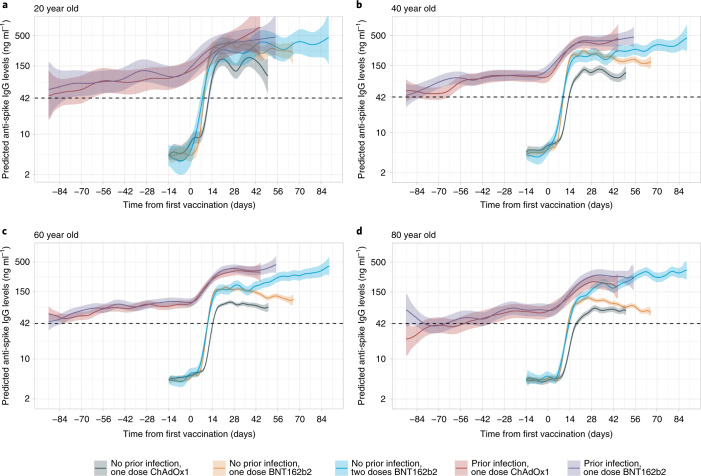
Predicted anti-spike IgG levels by time from first vaccination for comparisons of vaccine type and prior infection status by age. **a**–**d**, Predicted anti-spike IgG levels (mAb45 ng ml^–1^ equivalent units) by time from first vaccination based on data from 40,131 participants without prior infection and 5,834 participants with prior infection. Data shown for 20 year olds (**a**), 40 year olds (**b**), 60 year olds (**c**) and 80 year olds (**d**). Black dotted line indicates the threshold of IgG positivity (42 ng ml^–1^). Line colour indicates predicted response for the different vaccine type and prior infection status (full models shown in Extended Data Fig. 8, plotted by vaccine in Fig. 3). The 95% CIs are calculated by prediction ± 1.96 × standard error of the prediction. Data identical to Fig. 3, but Fig. 3 represent age rather than vaccine type. Source data

**Extended Data Fig. 8:**
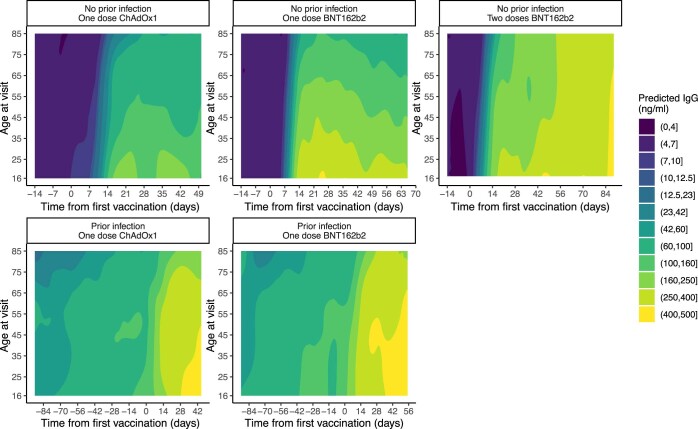
Predicted anti-spike IgG levels (ng/ml) by time from first vaccination and age based on data from 40,131 participants without prior infection and 5,834 participants with prior infection, according to vaccine type and prior infection status (full model). Predictions shown for specific ages in Fig. 3. Source data

In participants without evidence of prior infection, quantitative anti-spike IgG levels followed similar patterns to binary IgG positivity post-vaccination (Figs. 3 and 4 and Extended Data Fig. 8). Following a single dose of BNT162b2 or ChAdOx1, older participants reached lower peak levels and levels rose more slowly than in those of younger ages. Anti-spike IgG levels were initially lower after a single dose of ChAdOx1 than after BNT162b2. For example, 28 days post-vaccine, the following IgG levels (in ng ml^–1^ equivalents, with 95% CIs in parentheses) were reported for ChAdOx1 and BNT162b2, respectively: 73 (65–81) and 113 (104–123) for 80 year olds; 94 (87–100) and 163 (153–175) for 60 year olds; 113 (99–129) and 236 (214–261); for 40 year olds; and 127 (94–171) and 334 (266–420) for 20 year olds (Supplementary Table 3). As context, in a prior study^15^, protection from re-infection began to rise at antibody titres of ~20 ng ml^–1^, and increased as antibody titres rose to ~250 ng ml^–1^, with 50% protection against any PCR-positive result (symptomatic or asymptomatic) achieved at titres of 28 ng ml^–1^. The rate of increase in antibody levels was also slightly slower following the ChAdOx1 vaccine. For example, the estimated mean time to reaching the threshold for antibody positivity after the first vaccine in 40 year olds was 10 days after receiving BNT162b2 but 14 days after receiving ChAdOx1 (Fig. 3). However, antibody levels gradually decreased from ~35 days post-vaccination in participants receiving a single dose of BNT162b2 (Fig. 3), while there was no evidence of decrease in those receiving a single ChAdOx1 dose up to 49 days post-vaccination. Hence, differences in mean antibody levels between single doses of the two vaccines attenuated over time, particularly at older ages (Supplementary Table 3).

For two doses of BNT162b2, high anti-spike IgG levels were achieved 28 days after the first vaccination regardless of age (Supplementary Table 3). The anti-spike IgG levels after receiving one dose of BNT162b2 compared with two doses were similar in younger ages but were substantially attenuated at older ages, with differences starting earlier after the first vaccine and attenuating more rapidly with increasing age (Fig. 4).

In participants with evidence of prior infection, while vaccination increased antibody levels at all ages, the absolute increases were more modest. Older previously infected participants had lower IgG levels compared with younger ages before and after vaccination (Fig. 3). There was no evidence of a difference in post-vaccine response after prior infection between those receiving BNT162b2 or ChAdOx1 (Figs. 3 and 4). At intermediate ages, antibody levels were significantly higher with a single dose following natural infection than with two BNT162b2 doses, whereas two doses achieved similar antibody levels to one dose following natural infection at younger and older ages.

### Latent class analysis of antibody trajectory in participants without prior infection

**Fig. 5:**
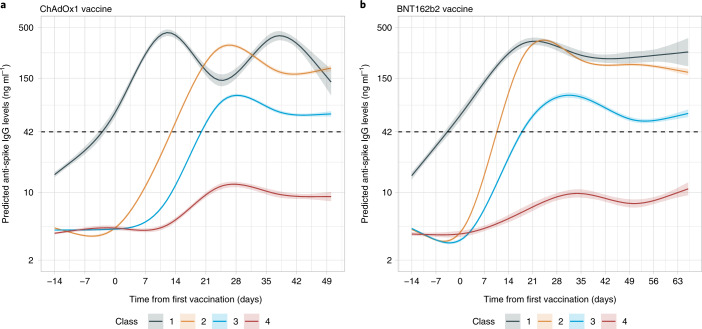
Predicted anti-spike IgG trajectory in participants without prior infection by class identified from latent class mixed models. Predicted anti-spike IgG trajectory in 36,518 participants without prior infection by class identified from latent class mixed models, using data from 14 days before vaccination to the 90th percentile of the observed time points after vaccination. **a**, One dose ChAdOx1 vaccine and no evidence of prior infection (*N* = 22,424 participants). **b**, One dose BNT162b2 vaccine and no evidence of prior infection (*N* = 14,094 participants). Black dotted line indicates the threshold of IgG positivity (42 ng ml^–1^). The 95% CIs are calculated by Monte Carlo approximation of the posterior distribution of the predicted values. The distribution of factors by class membership is shown in Supplementary Table 4. Class 1 = plausibly previously infected group (3.9% ChAdOx1, 3.9% BNT162b2), 2 = high-response group (31.6% ChAdOx1, 63.5% BNT162b2), 3 = medium-response group (58.7% ChAdOx1, 27.5% BNT162b2), 4 = low-response group (5.8% ChAdOx1, 5.1% BNT162b2). Source data

**Extended Data Fig. 9:**
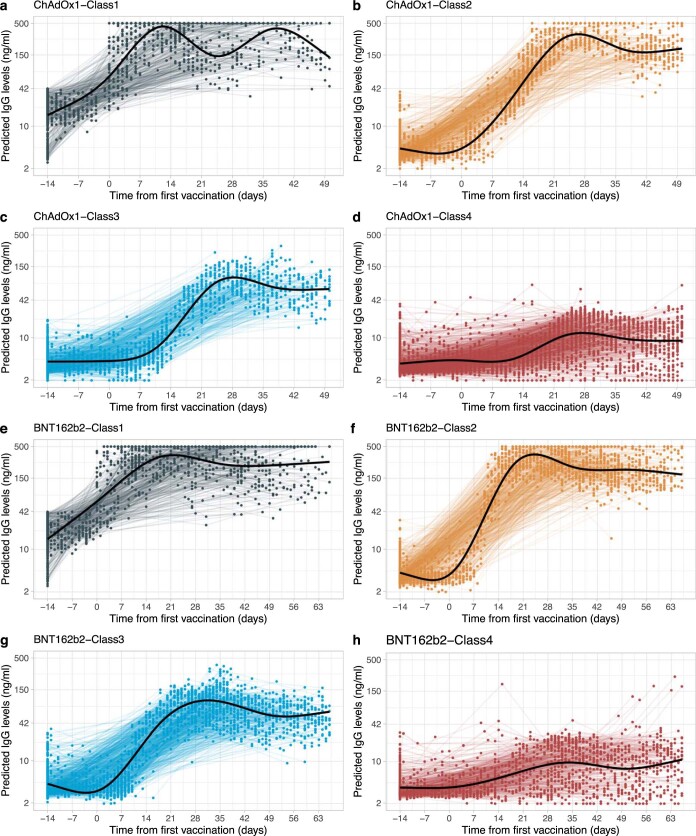
Individual trajectories by classes identified from latent class mixed models for single dose ChAdOx1 and BNT162b2 vaccine. In Class 1 and Class 4, all participants were plotted (ChAdOx1-Class 1: N = 867, Class 4: N = 1297. BNT162b2- Class 1: 547, Class 4: 720). In Class 2 and 3, 1,000 randomly selected participants were plotted for visualisation due to the large number. Colours: navy blue, Class 1; orange, Class 2; mid blue, Class 3; red, Class 4. Source data

**Extended Data Fig. 10:**
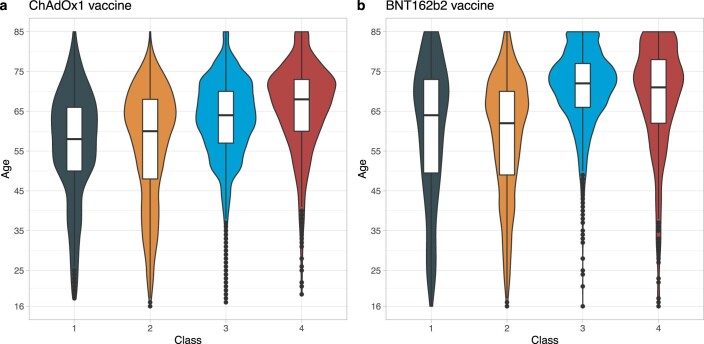
Age distribution by classes identified from latent class mixed models for single dose ChAdOx1 (N = 22,424) and BNT162b2 (N = 14,094) vaccines. 1 = ’plausibly previously infected’ group, 2 = ’high response’ group, 3 = ’medium response’ group, 4 = ’low response group’. Same area for each violin, see Supplementary Table 4 for numbers in each group. For the box and whisker inserts: centre line, median; box limits, upper and lower quartiles; whiskers, 1.5x interquartile range; points, outliers. Colours: navy blue, Class 1; orange, Class 2; mid blue, Class 3; red, Class 4. Source data

We used descriptive latent class mixed models to identify evidence for different subgroups of responses after vaccination. The best-fitting models identified four classes of antibody responses post-vaccination for both vaccines (Fig. 5, Extended Data Fig. 9 and Supplementary Table 4). In a ‘plausibly previously infected’ group (class 1, navy-blue line, comprising 3.9% of those receiving single-dose ChAdOx1 or BNT162b2), anti-spike IgG levels started higher pre-vaccination (but below the threshold for positivity) and rapidly rose. In a ‘high response’ group (class 2, orange line, 31.6% and 63.5% of ChAdOx1 and BNT162b2 recipients, respectively), IgG levels increased rapidly and to a higher level before plateauing. A ‘medium response’ group (class 3, mid-blue line, 58.7% and 27.5% of ChAdOx1 and BNT162b2 recipients) had mean antibody levels slightly below the high-response group but still above the positivity threshold. Last, participants in a ‘low response’ group (class 4, red line) had mean IgG levels below the positivity threshold throughout, peaking at ~10 ng ml^–1^, and their response was delayed. A similar percentage, 5.8% and 5.1% of participants receiving the ChAdOx1 or BNT162b2 vaccine, respectively, fell in this group. Low-response participants were older while high-response participants were younger for both vaccines (Extended Data Fig. 10). Low responders also had a higher proportion of males for the ChAdOx1 vaccine and people with long-term health conditions for both vaccines (*P* < 0.001). Many health conditions were more common in low responders (Supplementary Table 5), with taking immunosuppressants (aOR for class 4 versus class 2 or 3 responses, 3.91 [95% CI = 2.64–5.78]), rheumatoid arthritis (2.50 [1.66–3.76]), chronic liver disease (2.34 [1.06–5.19]), cancer (1.62 [1.31–1.99]), taking corticosteroids (1.59 [1.21–2.10]), type 2 diabetes (1.44 [1.07–1.93]), obesity (body–mass index ≥ 30 kg per m^2^, 1.25 [1.05–1.48]) and asthma (1.25 [1.03–1.52]) independently associated with low responses.

## Discussion

In this study based on 45,965 vaccinated participants from a large random sample of the UK population, we showed that post-vaccine anti-spike IgG responses vary by prior infection status, age, sex, vaccine type and number of doses received. In those who were previously infected, all age groups achieved high antibody responses after the first vaccination. In those without evidence of prior infection, older participants had lower and slower responses after the first vaccine dose than younger participants. Two vaccine doses achieved high responses across all age groups, and particularly increased the number of older people seroconverting to similar levels to those receiving one dose after prior infection, as recently reported in a smaller number of younger individuals^7^. A single dose of ChAdOx1 resulted in lower absolute antibody levels and slower responses compared with a single dose of BNT162b2. However, antibody levels after a single dose of BNT162b2 waned over time, whereas levels remained approximately constant after a single dose of ChAdOx1. Importantly, we did not identify a group who did not respond at all to vaccination; however, ~6% of participants were low responders to both vaccines, with low responses independently associated with several long-term health conditions.

The relative differences in vaccine response by age and previous infection status are similar to those reported by the REACT-2 study^12^ of binary point-of-care lateral flow assay results after a single dose of BNT162b2. However, our results showed a much higher antibody response than reported in REACT-2, especially in older people, despite studying the same UK population. These differences probably reflect the lower sensitivity of the assay used in REACT-2, despite efforts to adjust for this^12^. In our study, mean quantitative responses were not far from the positivity threshold, particularly for older age groups, which demonstrates the challenge in applying binary thresholds to what are essentially continuous data. This is particularly important given that the antibody levels required for protection are still unclear, with a study using the same assay as our study identifying a gradient of protection associated with quantitative antibody levels below the positivity threshold following previous infection^15^. Our study provides additional comparative data on antibody responses following the ChAdOx1 vaccine. Studies of healthcare workers also support an inverse association between antibody response and age in those receiving a single dose of the BNT162b2 vaccine^16,17^ or the ChAdOx1 vaccine^6^.

We found in those without evidence of previous infection, at older ages, females had a higher probability of being IgG positive post-vaccination than males, and females were more likely to be in the high-response latent class. Sex differences in antibody levels have also been described following natural infection^18^. These findings are consistent with observations that females generate stronger humoral immunity and greater vaccine efficacy than males^19,20^. However, a UK study of 3,610 healthcare workers (median age of 41 years) did not find any association between sex and single-dose ChAdOx1 or BNT162b2 antibody responses^6^, which is possibly explained by our finding that sex differences in antibody responses become more marked above 60 years of age.

Consistent with several previous studies^7,8,21^, we found that in previously infected participants, a single dose of ChAdOx1 or BNT162b2 led to high anti-spike IgG antibody positivity and quantitative levels. Where vaccine supplies are limited, this supports prioritizing those without evidence of previous infection for vaccination and, in particular, delaying or omitting second doses in those with robust serological evidence of previous infection in these scenarios. We also find that receiving two vaccine doses significantly increased seropositivity and antibody levels in older participants, but the short-term incremental increase in 20–40 year olds with a second vaccine was much smaller, thereby suggesting that older age groups should be prioritized for a second vaccination. However, protection from infection following seroconversion is not absolute^6^, with seroconversion rates after the first dose vaccination exceeding the reduction in symptomatic infection. Therefore, vaccine efficacy against clinical outcomes as well as antibody responses should contribute to prioritization decisions. In a related UK-wide study^22^ and a study from Israel^23^, high levels of protection from infection following natural infection were observed that were comparable to those seen after two doses of vaccination without prior infection. In the latter study^23^, the authors question whether previously infected individuals require vaccination; our data show that vaccination does boost antibody responses after previous infection, although the impact on protection from infection over varying timescales requires further study.

Our latent class analysis identified four distinct types of vaccine response. The low-response class was more common in older participants and those with long-term health conditions, but comprised a similar percentage receiving the different vaccines. Further follow-up is needed to identify whether the modest increases in antibody levels achieved still lead to some protection against key outcomes such as hospitalization, death or onward transmission, and to what extent second vaccine doses boost this initial suboptimal response. This low-responder group could be identified by a negative antibody result on our assay from day 28 post-vaccine. Similar underlying latent classes were identified following single doses of the two vaccines, with different mean responses overall due to different percentages estimated to fall into the high-response and medium-response classes for ChAdOx1 and BNT162b2. Further studies are also required to assess whether different degrees of response are associated with different rates of waning over time and protection against clinical outcomes. A recent study of 10,412 of long-term care residents showed 65% and 68% protection against laboratory-confirmed SARS-CoV-2 infection 28–42 days after vaccination with the ChAdOx1 and BNT162b2 vaccines, respectively, which suggests that differences in antibody responses may have a limited impact on outcomes, at least in the short term^24^. Similar short-term (6 weeks) protection against symptomatic infection, hospitalization and death with single doses of both vaccines was also seen in adults aged >70 years in England^25^.

Using data from all participants in the national survey^22^, we found a 76% reduction in symptomatic SARS-CoV-2 infection following a first vaccine dose, rising to 95% after two doses, with no evidence of differences after BNT162b2 and ChAdOx1 vaccinations. A major outstanding question is the extent to which antibody titres (and other immunological measurements) are correlates of post-vaccination protection and its duration. Pooled data from clinical trials and a post-infection study show a strong relationship between mean neutralization levels and reported protection^14^, estimating that protection from infection is likely to fall over 250 days (given an estimated 2-month half-life for post-vaccine neutralization titres), but with largely preserved protection from severe infection. In this current study, antibodies were measured in only a subset of survey participants, so data are currently insufficient to directly estimate the relationship between post-vaccine antibody titres and protection from infection. However, previous studies provide some indication. For example, using the same assay, post-infection antibody titres of 28 ng ml^–1^ mAb45 equivalents (or 7 million fluorescence units) were associated with 50% protection from any subsequent PCR-positive result in healthcare workers^15^. Most of those vaccinated in this study achieved levels of >28 ng ml^–1^, including at older ages, for example, mean 73 and 113 ng ml^–1^ for 80 year olds 28 days after the first ChAdOx1 or BNT162b2 dose, respectively. Only ~6% of individuals were in the low-responder latent class, with peak antibody levels reaching only ~10 ng ml^–1^.

Study limitations include currently insufficient data to analyse responses following two doses of ChAdOx1 (533 participants without and 66 with prior infection). Data on antibody responses between 8 and 12 weeks after the first dose without a second dose were also limited. Further follow-up will be required to assess the duration of responses to all vaccines and how variations in the interval between first and second doses affects this. Although our study is representative of those vaccinated to date in the UK, vaccination prioritization means that we have fewer data on healthy younger adults. We show that multiple long-term health conditions are associated with lower antibody responses, but additional studies are required to understand their significance for vaccine protection. Our study assesses responses using a single assay; however, responses were calibrated to a monoclonal antibody such that these can be readily compared with other studies. Neutralizing antibody and T-cell responses were not assayed in this study. However, a recent much smaller study of T-cell responses post-vaccination in healthcare workers found qualitatively similar findings^26^.

In summary, in this population-representative study of individuals vaccinated to date in the UK, vaccination results in detectable SARS-CoV-2 anti-spike IgG levels in the majority of individuals after first vaccination. High rates of seroconversion and high quantitative antibody levels following one dose of vaccine after previous infection and in younger previously uninfected individuals potentially supports single dose or delayed second dose vaccination in these groups if vaccine supplies are limited, although the potential for this to lead to antigenic evolution requires investigation^27^. Further data from this study and others will be needed to assess the extent to which quantitative antibody levels can be used as a correlate of vaccine-mediated protection.

## Methods

### Population and data

We used data from the UK’s Office for National Statistics (ONS) COVID-19 Infection Survey (ISRCTN21086382) from 26 April 2020 to 6 April 2021. The survey randomly selects private households on a continuous basis from address lists and previous surveys conducted by the ONS or the Northern Ireland Statistics and Research Agency to provide a representative sample across the four countries constituting the UK (England, Wales, Northern Ireland and Scotland). Following verbal agreement to participate, a study worker visited each household to take written informed consent. This consent was obtained from parents/carers for those aged 2–15 years, while those aged 10–15 years also provided written assent. Children aged <2 years were not eligible for the study. At the first visit, participants were asked for (optional) consent for follow-up visits every week for the next month, then monthly for 12 months from enrolment. For a random 10% of households, those aged ≥16 years were invited to provide blood monthly for serological testing from enrolment. Nose and throat swabs were taken from all consenting household members, according to the follow-up schedule they agreed to at enrolment. Any individual aged ≥16 years from a household where anyone tested positive on a nose and throat swab was also invited to provide blood at all subsequent monthly visits. Participants provided survey data on sociodemographic characteristics and vaccination status. Details on the sampling design are provided elsewhere^28^. The study protocol is available at https://www.ndm.ox.ac.uk/covid-19/covid-19-infection-survey/protocol-and-information-sheets. The study received ethical approval from the South Central Berkshire B Research Ethics Committee (20/SC/0195).

Vaccination data were reported by participants to the COVID-19 Infection Survey and obtained from linkage to the National Immunisation Management Service, which holds a database of all individuals vaccinated in the National Health Service COVID-19 vaccination programme in England. Similar linked administrative data were not available for Northern Ireland, Scotland or Wales. Information on the date, doses and type of vaccination were included in the dataset.

Only participants who received at least one dose of the ChAdOx1 or BNT162b2 vaccine were included; other vaccines were very rarely reported. Participants aged ≥16 years who had received at least one dose of vaccine from 8 December 2020 onwards with one or more antibody measurements from 91 days before their first vaccination date up until 6 April 2021 were included.

### Laboratory testing

SARS-CoV-2 antibody levels were measured using an ELISA detecting anti-trimeric spike IgG developed by the University of Oxford^28,29^. All testing was performed at the University of Oxford. Normalized results are reported in ng ml^–1^ of mAb45 monoclonal antibody equivalents. Up to 26 February 2021, the assay was performed using a fluorescence detection mechanism as previously described^29^, using a threshold of 8 million units to identify positive samples. Subsequent testing was performed with a CE-marked version of the assay, the Thermo Fisher OmniPATH 384 Combi SARS-CoV-2 IgG ELISA, which uses the same antigen, with a colorimetric detection system. mAb45 is the manufacturer-provided monoclonal antibody calibrant for this quantitative assay. To allow conversion of fluorometrically determined values in arbitrary units, 3,840 samples were run in parallel on both systems and compared. A piece-wise linear regression was used to generate the following conversion formula: log_10_(mAb45 units) = 0.221738 + 1.751889 × 10^−7^ × fluorescence units + 5.416675 ×10^−7^ × (fluorescence units > 9,190,310) × (fluorescence units − 9,190,310).

A threshold of ≥42 ng ml^–1^ was used to identify IgG-positive samples, corresponding to the 8 million units with fluorescence detection. In this analysis, measurements <2 ng ml^–1^ (395 observations, 0.4%) and >500 ng ml^–1^ (7,707 observations, 7%) were truncated at 2 and 500 ng ml^–1^, respectively.

PCR assays of combined nose and throat swabs were undertaken using the Thermo Fisher TaqPath SARS-CoV-2 assay at high-throughput national ‘Lighthouse’ laboratories in Glasgow and Milton Keynes (up until 8 February 2021). PCR outputs were analysed using UgenTec FastFinder 3.300.5, with an assay-specific algorithm and decision mechanism that allows conversion of amplification assay raw data into test results with minimal manual intervention. Samples are called positive if at least a single N-gene and/or ORF1ab are detected (although S-gene cycle threshold (Ct) values are determined, S-gene detection alone is not considered sufficient to call a sample positive^28^) and PCR traces exhibit an appropriate morphology.

### Statistical analysis

Participants with a SARS-CoV-2 PCR-positive nose/throat swab or a prior positive anti-spike IgG antibody result at any time before vaccination were considered to have been previously infected with SARS-CoV-2, irrespective of whether they had reported previous symptoms or not. Regular PCR results from the survey were included in this classification, but not self-reported PCR or lateral flow test results obtained outside the study. We used multivariable logistic and linear generalized additive models to investigate binary (positive/negative) and quantitative (log_10_(mAb45 units)) anti-spike IgG antibody measurements after the first vaccination. Given the prior hypothesis that response to vaccination would vary differentially by age and time according to vaccine type and prior infection, we built separate models by vaccine type, for those receiving one or two vaccinations, and by prior infection status. For participants receiving one vaccine dose, four models were fitted, for each vaccine and in those with and without evidence of prior infection. Two-dose models were only fitted for those receiving BNT162b2 without evidence of prior infection, as the sample sizes were small for other groups: 315 participants with prior infection receiving two BNT162b2 doses; 533 participants without prior infection receiving two ChAdOx1 doses; 66 participants with prior infection receiving two ChAdOx1 doses (Extended Data Fig. 1).

Models were adjusted for participant age using a tensor product of B-splines to allow for nonlinearity and interaction between age and time since vaccination. The smoothing penalty was selected using fast restricted maximum likelihood as implemented in the mcgv R package. We included a random intercept for each participant to account for repeated measurements using a random effect smoother with the number of basis functions equal to the number of participants. The 95% CIs were calculated using the following formula: prediction ± 1.96 × standard error of prediction. The date of the first vaccination was set as *t* = 0. For those with no prior evidence of infection, we truncated time at *t* = 0 and *t* = −14 for logistic and linear models, respectively (*t* = −14 for linear models to estimate IgG baseline pre-vaccination). We excluded measurements taken after the 90th percentile of observed time points for all models to avoid undue influence from outliers at late time points. Any participant receiving a second BNT162b2 dose after the 90th percentile for the single BNT162b2 dose group (61 days) was censored at this time point and included in the one-dose group (1,383 (3%) participants were censored in this way). Age was truncated at 85 years in all analyses to avoid outlier influence.

To investigate predictors of antibody response in those without prior evidence of infection, we considered the latest antibody measurement per participant between 14 and 60 days after the first vaccine. We used multivariable logistic regression to examine the association between antibody positivity and vaccine type and doses received by this measurement time, demographic factors (age, sex and ethnicity), household size, deprivation ranking (index of multiple deprivation in England and equivalent percentile ranking in Wales, Northern Ireland and Scotland), whether the participant reported working in patient-facing healthcare or social care, whether they reported working in a care home (any role), and whether they reported having a long-term health condition. Nonlinearity in age was accounted for using restricted natural cubic splines with internal knots at the 20th, 40th, 60th and 80th percentiles of unique values, and boundary knots at 5th and 95th percentiles. We tested for and added interactions between age and other variables if the interaction *P* value was <0.05.

For those without evidence of prior infection who received a single dose of vaccine, we also investigated whether we could identify distinct patterns of antibody responses, using latent class mixed models to identify subgroups with different antibody trajectories after the first vaccination. Natural cubic splines (internal knots at the 20th, 40th, 60th and 80th percentiles of unique values, and boundary knots at 5th and 95th percentiles) were used to model time since vaccination as a fixed effect and a random intercept was added to account for individual variability. Within-class between-individual heterogeneity may also be present in the trajectories; however, models accounting for random slopes failed to converge. Age with natural cubic splines (same as above), sex, reported long-term health conditions and whether the participant was a healthcare worker were included as covariates for class membership. The 95% CI of the estimation was calculated by a Monte Carlo approximation of the posterior distribution of the predicted values, using the 2.5% and 97.5% percentiles. The number of classes was determined by minimizing the Bayesian information criterion for each vaccine, and then fitting the maximum number of classes (four) to both groups for comparability.

To compare prevalence of long-term health conditions across different subgroups identified, participants from England were linked to the General Practice Extraction Service Data for Pandemic Planning and Research via their NHS number (equivalent data not available for participants from the Devolved Administrations (Wales, Northern Ireland and Scotland)). A range of pre-existing conditions across organ systems were identified from diagnosis codes over the 10-year look-back period 1 January 2010 to 24 January 2020 (the date of the first known case of COVID-19 in the UK). Body–mass index was the most recently recorded measurement over the look-back period, without imputation. Participants were recorded as being on antihypertensive medication, diabetes medication, corticosteroids or immunosuppressants if they were prescribed these treatments within 90 days of the end of the look-back period. All clinical variables were derived from primary care records only; hospital admissions data were not used.

Analyses were performed using the tidyverse (v.1.3.0), mgcv (v.1.8-31), splines (v.3.6.1), lcmm (v.1.9.2), ggeffects (v.0.14.3), sandwich (v.3.0-0), arsenal (v.3.4.0), emmeans (v.1.5.1), cowplot (v.1.1.0), gmodels (v.2.18.1) and mgcViz (v.0.1.6)) libraries in R (v.3.6). Model diagnostics used residual checks for generalized additive models, including distributions and quantile–quantile plots using check.gamViz, which showed normally distributed residuals but with some skew due to the assay upper limit of 500 ng ml^–1^. For the logistic model for antibody response 14–60 days post-vaccination, the *C*-statistic showed modest discriminatory power (0.66), but there was no evidence of misspecification (Homer–Lemeshow *P* = 1.00).

### Reporting Summary

Further information on research design is available in the Nature Research Reporting Summary linked to this article.

## Supplementary information

Supplementary Information Supplementary Tables 1–5. Supplementary Table 1: Participants’ characteristics by cohort. Supplementary Table 2: Predicted percentage probability of anti-spike IgG seropositivity with 95% CI according to age, vaccine type and dose, and prior infection status by weeks after vaccination. Supplementary Table 3: Predicted anti-spike IgG levels (ng ml^–1^) with 95% CI according to age, vaccine type and dose, and prior infection status by weeks after vaccination. Supplementary Table 4: Characteristics of classes identified from latent class mixed models for single dose ChAdOx1 and BNT162b2 vaccines in those without evidence of prior infection. Supplementary Table 5: Previous health conditions identified from linked primary care records in 29,575 participants from England without evidence of prior infection.

Reporting Summary

## Data Availability

Data are still being collected for the COVID-19 Infection Survey. De-identified study data are available for access by accredited researchers in the ONS Secure Research Service (SRS) for accredited research purposes under part 5, chapter 5 of the Digital Economy Act 2017. For further information about accreditation, contact Research.Support@ons.gov.uk or visit the SRS website. Source data are provided with this paper.

## Source data

Source Data Fig. 1 Statistical source data.
Source Data Fig. 2 Statistical source data.
Source Data Fig. 3 Statistical source data.
Source Data Fig. 4 Statistical source data.
Source Data Fig. 5 Statistical source data.
Source Data Extended Data Fig. 2 Statistical source data.
Source Data Extended Data Fig. 3 Statistical source data.
Source Data Extended Data Fig. 4 Statistical source data.
Source Data Extended Data Fig. 5 Statistical source data.
Source Data Extended Data Fig. 6 Statistical source data.
Source Data Extended Data Fig. 7 Statistical source data.
Source Data Extended Data Fig. 8 Statistical source data.
Source Data Extended Data Fig. 9 Statistical source data.
Source Data Extended Data Fig. 10 Statistical source data.

## Code availability

A copy of the analysis code is available at https://github.com/jiaweioxford/COVID19_vaccine_antibody_response.

## References

[1] Medicines and Healthcare products Regulatory Agency. *Regulatory Approval of Pfizer/BioNTech Vaccine for COVID-19* (GOV.UK, 2020); https://www.gov.uk/government/publications/regulatory-approval-of-pfizer-biontech-vaccine-for-covid-19

[2] Medicines and Healthcare products Regulatory Agency. *Oxford University/AstraZeneca COVID-19 Vaccine Approved* (GOV.UK, 2020); https://www.gov.uk/government/news/oxford-universityastrazeneca-covid-19-vaccine-approved

[3] *UK COVID-19 Vaccines Delivery Plan Contents* (Department of Health and Social Care, 2021).

[4] Polack FP (2020). Safety and efficacy of the BNT162b2 mRNA Covid-19 vaccine. N. Engl. J. Med..

[5] Voysey M (2021). Safety and efficacy of the ChAdOx1 nCoV-19 vaccine (AZD1222) against SARS-CoV-2: an interim analysis of four randomised controlled trials in Brazil, South Africa, and the UK. Lancet.

[6] Eyre, D. W. et al. Quantitative SARS-CoV-2 anti-spike responsesto Pfizer–BioNTech and Oxford–AstraZeneca vaccines by previous infection status. *Clin. Microbiol. Infect.*10.1016/j.cmi.2021.05.041 (2021). 10.1016/j.cmi.2021.05.041 PMC8180449 34111577

[7] Ebinger JE (2021). Antibody responses to the BNT162b2 mRNA vaccine in individuals previously infected with SARS-CoV-2. Nat. Med..

[8] Ciccone, E. J. et al. SARS-CoV-2 seropositivity after infection and antibody response to mRNA-based vaccination. Preprint at *medRxiv*10.1101/2021.02.09.21251319 (2021).

[9] Saadat S (2021). Binding and neutralization antibody titers after a single vaccine dose in health care workers previously infected with SARS-CoV-2. JAMA.

[10] Subbarao S (2021). Robust antibody responses in 70–80-year-olds 3 weeks after the first or second doses of Pfizer/BioNTech COVID-19 vaccine, United Kingdom, January to February 2021. Euro Surveill..

[11] Parry, H. M. et al. BNT162b2 vaccination in people over 80 years of age induces strong humoral immune responses with cross neutralisation of P.1 Brazilian variant. Preprint at *SSRN*10.2139/ssrn.3816840 (2021). 10.7554/eLife.69375 PMC8500710 34586068

[12] Ward, H. et al. REACT-2 Round 5: increasing prevalence of SARS-CoV-2 antibodies demonstrate impact of the second wave and of vaccine roll-out in England. Preprint at *medRxiv*10.1101/2021.02.26.21252512 (2021).

[13] Harvala H (2021). Convalescent plasma therapy for the treatment of patients with COVID-19: assessment of methods available for antibody detection and their correlation with neutralising antibody levels. Transfus. Med..

[14] Khoury DS (2021). Neutralizing antibody levels are highly predictive of immune protection from symptomatic SARS-CoV-2 infection. Nat. Med..

[15] Lumley SF (2021). Antibody status and incidence of SARS-CoV-2 infection in health care workers. N. Engl. J. Med..

[16] Abu Jabal K (2021). Impact of age, ethnicity, sex and prior infection status on immunogenicity following a single dose of the BNT162b2 mRNA COVID-19 vaccine: real-world evidence from healthcare workers, Israel, December 2020 to January 2021. Euro Surveill..

[17] Prendecki M (2021). Effect of previous SARS-CoV-2 infection on humoral and T-cell responses to single-dose BNT162b2 vaccine. Lancet.

[18] Grzelak, L. et al. Sex differences in the evolution of neutralizing antibodies to SARS-CoV-2. *J. Infect. Dis.*10.1093/infdis/jiab127 (2021). 10.1093/infdis/jiab127 PMC7989436 33693749

[19] Chang WH (2020). A review of vaccine effects on women in light of the COVID-19 pandemic. Taiwan. J. Obstet. Gynecol..

[20] Klein SL, Flanagan KL (2016). Sex differences in immune responses. Nat. Rev. Immunol..

[21] Krammer F (2021). Antibody responses in seropositive persons after a single dose of SARS-CoV-2 mRNA vaccine. N. Engl. J. Med..

[22] Pritchard, E. et al. Impact of vaccination on new SARS-CoV-2 infections in the United Kingdom. *Nat. Med.*10.1038/s41591-021-01410-w (2021). 10.1038/s41591-021-01410-w PMC8363500 34108716

[23] Goldberg, Y. et al. Protection of previous SARS-CoV-2 infection is similar to that of BNT162b2 vaccine protection: a three-month nationwide experience from Israel. Preprint at *medRxiv*10.1101/2021.04.20.21255670 (2021).

[24] Shrotri, M. et al. Vaccine effectiveness of the first dose of ChAdOx1 nCoV-19 and BNT162b2 against SARS-CoV-2 infection in residents of long-term care facilities in England (VIVALDI): a prospective cohort study. *Lancet Infect. Dis.*10.1016/S1473-3099(21)00289-9 (2021). 10.1016/S1473-3099(21)00289-9 PMC8221738 34174193

[25] Bernal, J. L. et al. Effectiveness of the Pfizer-BioNTech and Oxford-AstraZeneca vaccines on covid-19 related symptoms, hospital admissions, and mortality in older adults in England: test negative case-control study. *BMJ*10.1136/bmj.n1088 (2021). 10.1136/bmj.n1088 PMC8116636 33985964

[26] Angyal, A. et al. T-cell and antibody responses to first BNT162b2 vaccine dose in previously SARS-CoV-2-infected and infection-naive UK healthcare workers: a multicentre, prospective, observational cohort study. Preprint at *SSRN*10.2139/ssrn.3812375 (2021).

[27] Saad-Roy CM (2021). Epidemiological and evolutionary considerations of SARS-CoV-2 vaccine dosing regimes. Science.

[28] Pouwels KB (2021). Community prevalence of SARS-CoV-2 in England from April to November, 2020: results from the ONS Coronavirus Infection Survey. Lancet Public Health.

[29] Ainsworth M (2020). Performance characteristics of five immunoassays for SARS-CoV-2: a head-to-head benchmark comparison. Lancet Infect. Dis..

