# Supplementary material for: Antibody responses to SARS-CoV-2 vaccines in 45,965 adults from the general population of the United Kingdom
Source: Nat Microbiol. 2021 Jul 21;6(9):1140–9. doi: 10.1038/s41564-021-00947-3 (PMC8294260; doi:10.1038/s41564-021-00947-3)
Supplement: Supplementary file 1 — Supplementary Tables 1–5. Supplementary Table 1: Participants’ characteristics by cohort. Supplementary Table 2: Predicted percentage probability of anti-spike IgG seropositivity with 95% CI according to age, vaccine type and dose, and prior infection status by weeks after vaccination. Supplementary Table 3: Predicted anti-spike IgG levels (ng ml–1) with 95% CI according to age, vaccine type and dose, and prior infection status by weeks after vaccination. Supplementary Table 4: Characteristics of classes identified from latent class mixed models for single dose ChAdOx1 and BNT162b2 vaccines in those without evidence of prior infection. Supplementary Table 5: Previous health conditions identified from linked primary care records in 29,575 participants from England without evidence of prior infection. [file 41564_2021_947_MOESM1_ESM.pdf]

nature portfolio

https://doi.org/10.1038/s41564-021-00947-3

Supplementary information

Antibody responses to SARS-CoV-2 vaccines in 45,965 adults from the general population of the United Kingdom

In the format provided by the
authors and unedited

The impact of SARS-CoV-2 vaccines on antibody responses in the general population in the United Kingdom: Supplementary tables 1-5

	No evidence of prior infection			Evidence of prior infection		Total (N=45965)	p value
	One dose ChAdOx1 (N=23368)	One dose BNT162b2 (N=14894)	Two doses BNT162b2 (N=1869)	One dose ChAdOx1 (N=3767)	One dose BNT162b2 (N=2067)		
Duration between two doses [Median (IQR)]			31 (21-47)				
Age							< 0.001
Median	63	66	70	61	62	64	
IQR	55, 70	54, 73	53, 80	52, 68	50, 70	54, 71	
Sex							< 0.001
Female	12510 (53.5%)	8497 (57.0%)	1153 (61.7%)	2034 (54.0%)	1136 (55.0%)	25330 (55.1%)	
Male	10858 (46.5%)	6397 (43.0%)	716 (38.3%)	1733 (46.0%)	931 (45.0%)	20635 (44.9%)	
Ethnicity							< 0.001
Non-white	1110 (4.8%)	817 (5.5%)	118 (6.3%)	241 (6.4%)	148 (7.2%)	2434 (5.3%)	
White	22258 (95.2%)	14077 (94.5%)	1751 (93.7%)	3526 (93.6%)	1919 (92.8%)	43531 (94.7%)	
Household size							< 0.001
1	5019 (21.5%)	3302 (22.2%)	501 (26.8%)	721 (19.1%)	395 (19.1%)	9938 (21.6%)	
2	12990 (55.6%)	8260 (55.5%)	985 (52.7%)	1916 (50.9%)	1072 (51.9%)	25223 (54.9%)	
3	2863 (12.3%)	1688 (11.3%)	168 (9.0%)	545 (14.5%)	312 (15.1%)	5576 (12.1%)	
4	1797 (7.7%)	1130 (7.6%)	145 (7.8%)	403 (10.7%)	202 (9.8%)	3677 (8.0%)	
5+	699 (3.0%)	514 (3.5%)	70 (3.7%)	182 (4.8%)	86 (4.2%)	1551 (3.4%)	
Deprivation percentile							< 0.001
Median	63	64	64	60	57	63	
IQR	40, 82	40, 83	38, 83	35, 81	32, 79	39, 82	
Report working in patient facing healthcare							< 0.001
No	22860 (97.8%)	13430 (90.2%)	1466 (78.4%)	3636 (96.5%)	1828 (88.4%)	43220 (94.0%)	
Yes	508 (2.2%)	1464 (9.8%)	403 (21.6%)	131 (3.5%)	239 (11.6%)	2745 (6.0%)	
Report working in person facing social care							< 0.001
No	23149 (99.1%)	14548 (97.7%)	1800 (96.3%)	3717 (98.7%)	2014 (97.4%)	45228 (98.4%)	
Yes	219 (0.9%)	346 (2.3%)	69 (3.7%)	50 (1.3%)	53 (2.6%)	737 (1.6%)	
Report working in a care home (any role)							< 0.001
No	23195 (99.3%)	14551 (97.7%)	1778 (95.1%)	3701 (98.2%)	2011 (97.3%)	45236 (98.4%)	
Yes	173 (0.7%)	343 (2.3%)	91 (4.9%)	66 (1.8%)	56 (2.7%)	729 (1.6%)	
Report having long-term health condition							< 0.001
No	15774 (67.5%)	9597 (64.4%)	1217 (65.1%)	2645 (70.2%)	1398 (67.6%)	30631 (66.6%)	
Yes	7594 (32.5%)	5297 (35.6%)	652 (34.9%)	1122 (29.8%)	669 (32.4%)	15334 (33.4%)	

Supplementary Table 1. Participants' characteristics by cohort. Continuous variables were compared using Kruskall-Wallis tests, and categorical variables were compared using Chi-squared tests. Note: higher deprivation percentile means living in a less deprived area.

		No prior infection			Prior infection	
Time (days)	Age	ChAdOx1 one dose	BNT162b2 one dose	BNT162b2 two doses	ChAdOx1 one dose	BNT162b2 one dose
Logistic model		Predicted probability of anti-spike IgG positivity (95%CI)				
0	20	1 (0-3)	0 (0-1)	5 (1-16)	90 (82-95)	94 (88-97)
0	40	1 (0-1)	0 (0-1)	2 (1-5)	85 (80-88)	89 (84-92)
0	60	0 (0-0)	0 (0-0)	1 (1-2)	78 (75-82)	81 (77-84)
0	80	0 (0-0)	0 (0-0)	0 (0-1)	70 (61-78)	69 (59-77)
7	20	9 (4-20)	29 (17-45)	60 (34-81)	91 (83-96)	97 (94-99)
7	40	6 (4-10)	13 (9-18)	36 (23-52)	88 (83-91)	94 (90-96)
7	60	5 (4-6)	6 (4-8)	18 (12-25)	82 (78-85)	87 (83-90)
7	80	2 (1-4)	3 (2-5)	8 (4-14)	74 (66-81)	73 (64-81)
14	20	79 (62-90)	97 (92-99)	95 (87-98)	93 (86-97)	99 (97-100)
14	40	63 (55-71)	95 (92-97)	89 (80-94)	92 (89-94)	97 (95-99)
14	60	49 (44-53)	89 (86-91)	78 (70-83)	89 (87-92)	95 (93-96)
14	80	20 (14-28)	66 (59-72)	60 (49-70)	83 (76-88)	90 (84-93)
21	20	90 (77-96)	98 (94-99)	98 (93-99)	96 (89-98)	99 (97-100)
21	40	85 (78-90)	96 (94-98)	96 (91-98)	95 (92-97)	99 (97-99)
21	60	81 (77-84)	93 (91-95)	91 (87-94)	95 (93-96)	98 (97-98)
21	80	60 (52-68)	81 (75-85)	84 (77-89)	90 (85-93)	96 (93-98)
28	20	90 (74-96)	97 (93-99)	97 (89-99)	97 (92-99)	99 (97-100)
28	40	84 (76-89)	95 (92-97)	95 (90-98)	97 (94-98)	99 (98-100)
28	60	79 (75-83)	91 (89-93)	93 (89-95)	96 (95-97)	98 (97-99)
28	80	74 (66-80)	85 (80-89)	89 (84-93)	94 (90-96)	98 (95-99)
35	20	94 (79-99)	98 (95-99)	93 (83-98)	99 (93-100)	99 (97-100)
35	40	86 (78-91)	96 (93-97)	94 (88-97)	98 (95-99)	99 (98-100)
35	60	80 (75-84)	91 (89-93)	94 (91-96)	97 (96-98)	98 (97-99)
35	80	74 (67-80)	81 (76-85)	94 (90-97)	95 (92-97)	97 (95-99)
42	20	92 (72-98)	98 (94-99)	95 (86-98)	99 (93-100)	100 (98-100)
42	40	84 (75-90)	96 (93-97)	94 (88-97)	99 (95-100)	99 (98-100)
42	60	79 (73-84)	91 (88-93)	93 (89-95)	97 (95-99)	98 (97-99)
42	80	75 (67-81)	82 (77-86)	91 (85-95)	96 (91-98)	96 (93-98)
49	20	80 (39-96)	98 (94-99)	95 (87-98)	-	100 (98-100)
49	40	84 (71-92)	95 (92-97)	94 (89-97)	-	100 (97-100)
49	60	78 (67-86)	89 (85-92)	93 (89-95)	-	98 (96-99)
49	80	78 (67-86)	79 (74-84)	91 (85-94)	-	92 (86-96)

Supplementary Table 2. Predicted percentage probability of anti-spike IgG seropositivity with 95% confidence interval (CI) according to age, vaccine type and dose, and prior infection status by weeks after vaccination. The 95% confidence intervals are calculated by prediction ± 1.96*standard error of prediction.

		No prior infection			Prior infection	
**Time (days)**	**Age**	**ChAdOx1 one dose**	**BNT162b2 one dose**	**BNT162b2 two doses**	**ChAdOx1 one dose**	**BNT162b2 one dose**
**Linear model**		**Predicted anti-spike IgG levels (ng/ml) (95%CI)**				
0	20	7 (5-9)	4 (3-6)	5 (3-8)	123 (82-185)	139 (90-216)
0	40	6 (5-7)	5 (5-6)	5 (4-7)	96 (78-119)	126 (102-156)
0	60	5 (5-6)	5 (4-5)	6 (5-7)	94 (81-108)	98 (83-115)
0	80	4 (4-5)	5 (4-5)	5 (4-6)	69 (53-90)	71 (55-92)
7	20	9 (7-12)	14 (11-18)	30 (17-52)	167 (111-251)	220 (141-342)
7	40	7 (7-8)	10 (9-11)	17 (13-22)	169 (136-210)	202 (162-250)
7	60	6 (6-6)	7 (7-8)	10 (8-12)	152 (132-176)	138 (117-163)
7	80	5 (4-5)	5 (5-5)	6 (5-8)	88 (67-117)	85 (66-108)
14	20	90 (71-115)	293 (228-375)	182 (110-301)	242 (155-376)	307 (204-464)
14	40	50 (45-56)	201 (181-225)	151 (117-196)	306 (243-386)	301 (244-371)
14	60	38 (36-40)	127 (119-137)	105 (86-128)	248 (214-287)	254 (217-298)
14	80	16 (15-19)	61 (56-67)	48 (39-58)	126 (96-166)	143 (113-181)
21	20	197 (152-257)	325 (258-409)	287 (173-478)	318 (197-514)	363 (243-542)
21	40	113 (100-127)	250 (225-277)	219 (171-280)	381 (299-485)	398 (324-489)
21	60	91 (85-97)	173 (162-185)	166 (136-202)	312 (267-365)	361 (307-424)
21	80	52 (47-58)	101 (93-110)	123 (103-147)	182 (141-236)	223 (174-286)
28	20	127 (94-171)	334 (266-420)	259 (153-440)	389 (237-639)	380 (245-591)
28	40	113 (99-129)	236 (214-261)	232 (179-301)	369 (286-477)	402 (324-497)
28	60	94 (87-100)	163 (153-175)	192 (158-232)	347 (291-413)	385 (328-452)
28	80	73 (65-81)	113 (104-123)	163 (136-196)	224 (173-290)	281 (221-356)
35	20	192 (135-274)	359 (278-464)	250 (157-400)	485 (274-860)	401 (250-644)
35	40	125 (108-144)	216 (193-242)	196 (157-246)	356 (267-476)	404 (321-508)
35	60	92 (85-100)	159 (148-171)	160 (133-193)	345 (283-420)	361 (305-429)
35	80	71 (64-79)	108 (99-117)	212 (180-251)	218 (165-288)	295 (234-372)
42	20	188 (123-288)	287 (221-373)	344 (215-551)	622 (251-1542)	424 (262-685)
42	40	93 (80-109)	202 (178-229)	244 (192-310)	412 (273-621)	411 (320-527)
42	60	86 (78-95)	146 (134-159)	191 (159-229)	335 (259-433)	353 (292-426)
42	80	74 (66-83)	94 (86-104)	175 (145-211)	200 (146-275)	280 (222-353)
49	20	107 (59-196)	269 (206-351)	382 (241-608)	-	450 (260-777)
49	40	108 (85-139)	177 (155-202)	264 (210-332)	-	441 (336-579)
49	60	79 (66-94)	117 (106-129)	218 (181-263)	-	385 (312-475)
49	80	73 (62-87)	81 (74-89)	220 (184-262)	-	273 (212-351)

Supplementary Table 3. Predicted anti-spike IgG levels (ng/ml) with 95% confidence interval (CI) according to age, vaccine type and dose, and prior infection status by weeks after vaccination. The 95% confidence intervals are calculated by prediction ± 1.96*standard error of prediction.

	ChAdOx1					BNT162b2				
	**Class 1 (N=867)**	**Class 2 (N=7097)**	**Class 3 (N=13163)**	**Class 4 (N=1297)**	**p value**	**Class 1 (N=547)**	**Class 2 (N=8951)**	**Class 3 (N=3876)**	**Class 4 (N=720)**	**p value**
Percentage	3.9%	31.6%	58.7%	5.8%		3.9%	63.5%	27.5%	5.1%	
Age					< 0.001					< 0.001
Median	58	60	64	68		64	62	72	71	
IQR	50, 66	48, 68	57, 70	60, 73		50, 73	49, 70	66, 77	62, 78	
Age group					< 0.001					< 0.001
16-34	73 (8.4%)	709 (10.0%)	167 (1.3%)	22 (1.7%)		63 (11.5%)	760 (8.5%)	12 (0.3%)	26 (3.6%)	
35-54	274 (31.6%)	1863 (26.3%)	2412 (18.3%)	165 (12.7%)		119 (21.8%)	2240 (25.0%)	231 (6.0%)	76 (10.6%)	
55-74	461 (53.2%)	4119 (58.0%)	9185 (69.8%)	862 (66.5%)		250 (45.7%)	4863 (54.3%)	2209 (57.0%)	354 (49.2%)	
>75	59 (6.8%)	406 (5.7%)	1399 (10.6%)	248 (19.1%)		115 (21.0%)	1088 (12.2%)	1424 (36.7%)	264 (36.7%)	
Sex					< 0.001					< 0.001
Female	453 (52.2%)	4467 (62.9%)	6505 (49.4%)	600 (46.3%)		305 (55.8%)	5577 (62.3%)	1721 (44.4%)	364 (50.6%)	
Male	414 (47.8%)	2630 (37.1%)	6658 (50.6%)	697 (53.7%)		242 (44.2%)	3374 (37.7%)	2155 (55.6%)	356 (49.4%)	
Report working in patient facing healthcare					< 0.001					< 0.001
No	834 (96.2%)	6902 (97.3%)	12966 (98.5%)	1276 (98.4%)		475 (86.8%)	7976 (89.1%)	3791 (97.8%)	692 (96.1%)	
Yes	33 (3.8%)	195 (2.7%)	197 (1.5%)	21 (1.6%)		72 (13.2%)	975 (10.9%)	85 (2.2%)	28 (3.9%)	
Report having long-term health condition					< 0.001					< 0.001
No	592 (68.3%)	5104 (71.9%)	8859 (67.3%)	636 (49.0%)		355 (64.9%)	6219 (69.5%)	2096 (54.1%)	344 (47.8%)	
Yes	275 (31.7%)	1993 (28.1%)	4304 (32.7%)	661 (51.0%)		192 (35.1%)	2732 (30.5%)	1780 (45.9%)	376 (52.2%)	
Posterior class-membership probability (%)					< 0.001					< 0.001
Median	98	82	71	99		94	89	86	100	
IQR	78, 100	61, 97	54, 92	84, 100		75, 100	74, 98	64, 97	91, 100	

Supplementary Table 4. Characteristics of classes identified from latent class mixed models for single dose ChAdOx1 and BNT162b2 vaccines in those without evidence of prior infection. Class 1='plausibly previously infected' group, 2='high response' group, 3='medium response' group, 4='low response group'. Continuous variables were compared using Kruskal-Wallis, and categorical variables were compared using Chi-squared tests.

Condition	Class 1	Class 2	Class 3	Class 4	Univariable OR (95% CI) Class 4 vs 2+3 combined	Univariable p value	Multivariable OR (95% CI) Class 4 vs 2+3 combined	Multivariable p value
**Total with primary care data**	**1121**	**13,057**	**13,808**	**1589**				
Angina	38 (3.4%)	336 (2.6%)	577 (4.2%)	95 (6.0%)	1.81 (1.45-2.25)	<0.0001	0.88 (0.49-1.58)	0.67
**Asthma**	**130 (11.6%)**	**1336 (10.2%)**	**1319 (9.6%)**	**198 (12.5%)**	**1.30 (1.11-1.51)**	**0.001**	**1.25 (1.03-1.52)**	**0.03**
Atrial fibrillation	42 (3.7%)	389 (3.0%)	578 (4.2%)	115 (7.2%)	2.09 (1.71-2.55)	<0.0001	1.16 (0.88-1.52)	0.28
**Cancer**	**54 (4.8%)**	**650 (5.0%)**	**970 (7.0%)**	**194 (12.2%)**	**2.17 (1.85-2.54)**	**<0.0001**	**1.62 (1.31-1.99)**	**<0.0001**
Chronic kidney disease	47 (4.2%)	491 (3.8%)	800 (5.8%)	178 (11.2%)	2.50 (2.12-2.95)	<0.0001	1.23 (0.98-1.54)	0.07
**Chronic liver disease**	**-**	**29 (0.2%)**	**30 (0.2%)**	**13 (0.8%)**	**3.75 (2.05-6.85)**	**<0.0001**	**2.34 (1.06-5.19)**	**0.04**
COPD	32 (2.9%)	289 (2.2%)	553 (4.0%)	99 (6.2%)	2.05 (1.66-2.55)	<0.0001	1.19 (0.90-1.58)	0.23
Coronary heart disease	50 (4.5%)	465 (3.6%)	762 (5.5%)	130 (8.2%)	1.86 (1.54-2.25)	<0.0001	1.03 (0.56-1.88)	0.92
Frailty	77 (6.9%)	670 (5.1%)	1109 (8.0%)	223 (14.0%)	2.30 (1.98-2.67)	<0.0001	1.18 (0.97-1.44)	0.11
Heart failure	10 (0.9%)	76 (0.6%)	185 (1.3%)	41 (2.6%)	2.70 (1.93-3.77)	<0.0001	1.16 (0.72-1.87)	0.54
Hypertension	211 (18.8%)	2436 (18.7%)	3351 (24.3%)	494 (31.1%)	1.64 (1.47-1.83)	<0.0001	1.03 (0.86-1.23)	0.77
Myocardial infarction	23 (2.1%)	187 (1.4%)	294 (2.1%)	50 (3.1%)	1.78 (1.33-2.40)	<0.0001	1.11 (0.66-1.87)	0.71
Osteoporosis	29 (2.6%)	317 (2.4%)	387 (2.8%)	73 (4.6%)	1.79 (1.40-2.29)	<0.0001	1.39 (1.01-1.91)	0.05
Peripheral arterial disease	-	77 (0.6%)	129 (0.9%)	30 (1.9%)	2.49 (1.69-2.66)	<0.0001	1.52 (0.95-2.43)	0.08
**Rheumatoid arthritis**	**-**	**88 (0.7%)**	**161 (1.2%)**	**85 (5.3%)**	**6.04 (4.70-7.77)**	**<0.0001**	**2.50 (1.66-3.76)**	**<0.0001**
Stroke	18 (1.6%)	128 (1.0%)	206 (1.5%)	31 (2.0%)	1.58 (1.09-2.29)	0.02	0.81 (0.49-1.35)	0.42
Transient ischaemic attack	14 (1.2%)	118 (0.9%)	197 (1.4%)	29 (1.8%)	1.57 (1.07-2.30)	0.02	0.91 (0.55-1.49)	0.70
Type 1 diabetes	14 (1.2%)	101 (0.8%)	67 (0.5%)	10 (0.6%)	1.01 (0.53-1.91)	0.98	-	-
**Type 2 diabetes**	**72 (6.4%)**	**848 (6.5%)**	**1227 (8.9%)**	**246 (15.5%)**	**2.19 (1.90-2.53)**	**<0.0001**	**1.44 (1.07-1.93)**	**0.02**
Overweight (BMI 25 to <30 kg/m^2)*	301 (41.6%)	2871 (35.7%)	3306 (38.2%)	355 (34.0%)	0.88 (0.77-1.00)	0.05	0.95 (0.81-1.12)	0.55
**Obese (BMI ≥30 kg/m^2)***	**177 (24.5%)**	**2264 (28.2%)**	**2580 (29.8%)**	**385 (36.9%)**	**1.43 (1.26-1.63)**	**<0.0001**	**1.25 (1.05-1.48)**	**0.01**
Antihypertensive medication	312 (27.8%)	3641 (27.9%)	5096 (36.9%)	763 (48.0%)	1.92 (1.73-2.12)	<0.0001	1.17 (0.97-1.41)	0.11
Diabetes medication	69 (6.2%)	812 (6.2%)	1108 (8.0%)	212 (13.3%)	2.00 (1.72-2.33)	<0.0001	1.10 (0.80-1.51)	0.56
**Corticosteroids**	**27 (2.4%)**	**325 (2.5%)**	**469 (3.4%)**	**120 (7.6%)**	**2.68 (2.20-3.27)**	**<0.0001**	**1.59 (1.21-2.10)**	**0.001**
**Immunosuppressants**	**10 (0.9%)**	**86 (0.7%)**	**178 (1.3%)**	**103 (6.5%)**	**6.98 (5.53-8.82)**	**<0.0001**	**3.91 (2.64-5.78)**	**<0.0001**

Supplementary Table 5. Previous health conditions identified from linked primary care records in 29,575 English participants without evidence of prior infection. Class 1='plausibly previously infected' group, 2='high response' group, 3='medium response' group, 4='low response group'. '-' indicates value not shown due to insufficient sample size. Overall linkage to the General Practice Extraction Service Data for Pandemic Planning and Research via the NHS number was achieved for 29,575 (93%) of 31,796 English participants (not available for participants from Wales, Scotland, or Northern Ireland). *Weights were available in 18,449 participants, with denominators by response Class 1-4 of 723, 8029, 8654, and 1043 respectively. Univariable ORs were estimated from all 28,454 participants in Classes 2-4. Multivariable ORs were estimated from a complete-case analysis of 18,288 participants in Classes 2-4 with known BMI (18,449) and no missing values for any other characteristics. Multivariable ORs are adjusted for: age (using a third-degree polynomial with knots at the 25th, 50th and 75th percentiles) interacted with sex; region (nine former Government Office Regions); coarse ethnic group (white or non-white); Index of Multiple Deprivation (IMD) quintile group; working in health or social care; and household size. The 95% confidence intervals are calculated by prediction ± 1.96*standard error of prediction; Wald p values are shown.
