# Supplementary material for: Antibody responses to SARS-CoV-2 vaccines in 45,965 adults from the general population of the United Kingdom
Source: Nat Microbiol. 2021 Jul 21;6(9):1140–9. doi: 10.1038/s41564-021-00947-3 (PMC8294260; doi:10.1038/s41564-021-00947-3)
Supplement: Supplementary file 2 — Reporting Summary [file 41564_2021_947_MOESM2_ESM.pdf]

nature research

Corresponding author(s): David Eyre

Last updated by author(s): Jun 22, 2021

Reporting Summary

Nature Research wishes to improve the reproducibility of the work that we publish. This form provides structure for consistency and transparency in reporting. For further information on Nature Research policies, see our Editorial Policies and the Editorial Policy Checklist.

Statistics

For all statistical analyses, confirm that the following items are present in the figure legend, table legend, main text, or Methods section.

n/a	Confirmed	
☐	☒	The exact sample size (*n*) for each experimental group/condition, given as a discrete number and unit of measurement
☐	☒	A statement on whether measurements were taken from distinct samples or whether the same sample was measured repeatedly
☐	☒	The statistical test(s) used AND whether they are one- or two-sided<br>*Only common tests should be described solely by name; describe more complex techniques in the Methods section.*
☐	☒	A description of all covariates tested
☐	☒	A description of any assumptions or corrections, such as tests of normality and adjustment for multiple comparisons
☐	☒	A full description of the statistical parameters including central tendency (e.g. means) or other basic estimates (e.g. regression coefficient) AND variation (e.g. standard deviation) or associated estimates of uncertainty (e.g. confidence intervals)
☐	☒	For null hypothesis testing, the test statistic (e.g. *F*, *t*, *r*) with confidence intervals, effect sizes, degrees of freedom and *P* value noted<br>*Give P values as exact values whenever suitable.*
☒	☐	For Bayesian analysis, information on the choice of priors and Markov chain Monte Carlo settings
☒	☐	For hierarchical and complex designs, identification of the appropriate level for tests and full reporting of outcomes
☐	☒	Estimates of effect sizes (e.g. Cohen's *d*, Pearson's *r*), indicating how they were calculated

Our web collection on statistics for biologists contains articles on many of the points above.

Software and code

Policy information about availability of computer code

Data collection	De-identified study data were accessed through the Office for National Statistics (ONS) Secure Research Service (SRS). The data available in SRS were prepared for data analysis using Stata MP 16.
Data analysis	All analyses were performed in R 3.6 using the following packages: tidyverse (version 1.3.0), mgcv (version 1.8-31), lcmm (version 1.9.2), ggeffects (version 0.14.3), sandwich (version 3.0-0), arsenal (version 3.4.0), emmeans (version 1.5.1), cowplot (version 1.1.0), gmodels (version 2.18.1). A copy of the analysis code is available at https://github.com/jiaweioxford/COVID19_vaccine_antibody_response.

For manuscripts utilizing custom algorithms or software that are central to the research but not yet described in published literature, software must be made available to editors and reviewers. We strongly encourage code deposition in a community repository (e.g. GitHub). See the Nature Research guidelines for submitting code & software for further information.

Data

Policy information about availability of data

All manuscripts must include a data availability statement. This statement should provide the following information, where applicable:

- Accession codes, unique identifiers, or web links for publicly available datasets
- A list of figures that have associated raw data
- A description of any restrictions on data availability

De-identified study data are available for access by accredited researchers in the ONS Secure Research Service (SRS) for accredited research purposes under part 5, chapter 5 of the Digital Economy Act 2017. Individuals can apply to be an accredited researcher using the short form on https://researchaccreditationservice.ons.gov.uk/ons/ONS_registration.ofml. Accreditation requires completion of a short free course on accessing the SRS. To request access to data in the SRS, researchers must submit a research project application for accreditation in the Research Accreditation Service (RAS). Research project applications are considered by the project team and the Research Accreditation Panel (RAP) established by the UK Statistics Authority at regular meetings. Project

application example guidance and an exemplar of a research project application are available. A complete record of accredited researchers and their projects is published on the UK Statistics Authority website to ensure transparency of access to research data. For further information about accreditation, contact Research.Support@ons.gov.uk or visit the SRS website.

Field-specific reporting

Please select the one below that is the best fit for your research. If you are not sure, read the appropriate sections before making your selection.

☒ Life sciences ☐ Behavioural & social sciences ☐ Ecological, evolutionary & environmental sciences

For a reference copy of the document with all sections, see nature.com/documents/nr-reporting-summary-flat.pdf

Life sciences study design

All studies must disclose on these points even when the disclosure is negative.

Sample size	45,965 participants aged ≥16 years from the general population of the United Kingdom who were first vaccinated between 8 December 2020 and 6 April 2021 contributed a total of 111,360 SARS-CoV-2 anti-spike IgG measurements taken at any point between 91 days before first vaccination date through to 6 April 2021. All available data were used for the current study, with the timing of the analysis determined by the duration of follow up available, rather than sample size given the number of participants in the study.
Data exclusions	No available data were excluded from the study.
Replication	All measurements and analytical assays were undertaken once. Serially collected samples from the same participants included demonstrate reproducibility over time.<br>The statistical analyses have been successfully replicated by two individuals.
Randomization	Recruitment randomised - we used data from the UK's Office for National Statistics (ONS) COVID-19 Infection Survey (CIS) (ISRCTN21086382) which randomly selects private households on a continuous basis from address lists and previous surveys conducted by the ONS or the Northern Ireland Statistics and Research Agency to provide a representative sample across the four countries comprising the UK (England, Wales, Northern Ireland, Scotland). No intervention.
Blinding	Not done, observational study

Reporting for specific materials, systems and methods

We require information from authors about some types of materials, experimental systems and methods used in many studies. Here, indicate whether each material, system or method listed is relevant to your study. If you are not sure if a list item applies to your research, read the appropriate section before selecting a response.

Materials & experimental systems

n/a	Involved in the study	
☐	☒	Antibodies
☒	☐	Eukaryotic cell lines
☒	☐	Palaeontology and archaeology
☒	☐	Animals and other organisms
☐	☒	Human research participants
☐	☒	Clinical data
☒	☐	Dual use research of concern

Methods

n/a	Involved in the study	
☒	☐	ChIP-seq
☒	☐	Flow cytometry
☒	☐	MRI-based neuroimaging

Antibodies

Antibodies used	The calibrant (mAb45) provided as part of the Thermo Fisher OmniPATH 384 Combi SARS-CoV-2 IgG ELISA kit is a monoclonal antibody. It is available as part of the test kit.
Validation	Details of the validation of the the Thermo Fisher OmniPATH 384 Combi SARS-CoV-2 IgG ELISA kit are provided in the manufacturer's instructions for use. The assay has also been validated in a head-to-head comparison of similar assays (The Lancet Infectious Diseases https://doi.org/10.1016/S1473-3099(20)30634-4), where the sensitivity was 99·1% (95%CI 97·8–99·7) and specificity was 99·0% (98·1–99·5).

Human research participants

Policy information about studies involving human research participants

Population characteristics	The median (IQR) age was 64 (54-71) years and 25,330 (55.1%) were female. 2,745 (6.0%) were healthcare workers, and 15,334 (33.4%) had a long-term health condition.
Recruitment	The Office for National Statistics (ONS) COVID-19 Infection Survey (CIS) is a large household survey with longitudinal follow-up (ISRCTN21086382, https://www.ndm.ox.ac.uk/covid-19/covid-19-infection-survey/protocol-and-information-sheets) (details in20). The study received ethical approval from the South Central Berkshire B Research Ethics Committee (20/SC/0195). Private households are randomly selected on a continuous basis from address lists and previous surveys to provide a representative sample across the UK. Following verbal agreement to participate, a study worker visited each selected household to take written informed consent for individuals aged 2 years and over. Parents or carers provided consent for those aged 2-15 years; those aged 10-15 years also provided written assent. All participants who completed the enrolment visit was offered a £50 voucher, and one £25 voucher for each further visit. For the current analysis we only included individuals aged 16 years and over.<br><br>While certain factors might drive non-response to invitations to participate, adjustment for covariates that may influence selection into the sample ensures that estimates of relative effects are not biased by factors that both influence selection into the sample and the risk of the outcome (model-based inference). We fit separate models to account for effect of vaccine type, number of vaccines received and prior infection. We adjust fitted models for age, including an interaction with time since vaccination to account for the age-stratified roll-out of vaccination in the United Kingdom.<br><br>In a logistic model of antibody positivity following first vaccine we adjust for other covariates including: demographic factors (age, sex, ethnicity), household size, deprivation ranking (index of multiple deprivation (IMD) in England and equivalent percentile ranking in Wales, Northern Ireland and Scotland), whether the participant reported working in patient-facing healthcare or social care, whether they reported working in a care home (any role), and whether they reported having a long-term health condition.<br><br>When fitting latent class models to antibody responses we adjust for age, sex, reported long-term health conditions, and whether the participant was a healthcare worker as covariates for class membership.
Ethics oversight	The study received ethical approval from the South Central Berkshire B Research Ethics Committee (20/SC/0195).

Note that full information on the approval of the study protocol must also be provided in the manuscript.

Clinical data

Policy information about clinical studies

All manuscripts should comply with the ICMJE guidelines for publication of clinical research and a completed CONSORT checklist must be included with all submissions.

Clinical trial registration	ISRCTN21086382
Study protocol	https://www.ndm.ox.ac.uk/covid-19/covid-19-infection-survey/protocol-and-information-sheets
Data collection	We used data from the UK's Office for National Statistics (ONS) COVID-19 Infection Survey (CIS) (ISRCTN21086382) from 26 April 2020 to 6 April 2021.
Outcomes	SARS-CoV-2 antibody levels were measured using an ELISA detecting anti-trimeric spike IgG developed by the University of Oxford (Thermo Fisher OmniPATH 384 Combi SARS-CoV-2 IgG ELISA).<br><br>For individuals vaccinated with the ChAdOx1 or BNT162b2 vaccines, we considered descriptive analyses of the relationship between time since first SARS-CoV-2 vaccination and binary and continuous anti-spike IgG antibody responses as co-primary outcomes. Separate analyses were performed by previous infection status and vaccine received, and age included as a covariate.<br><br>Secondary analyses included:<br>a) Determining associations between seroconversion 14-60 days post first vaccination in those not previously infected and age, sex, vaccine type, ethnicity, social deprivation, healthcare roles, and long-term health conditions<br>b) Descriptive latent class analyses to subdivide antibody responses following first vaccination in participants without prior infection. Separate models were fitted by vaccine type, and age, sex, reported long-term health conditions, and whether the participant was a healthcare worker were included as covariates for class membership.
